# Ultrasensitive ^129^Xe Magnetic Resonance Imaging: From Clinical Monitoring to Molecular Sensing

**DOI:** 10.1002/advs.202413426

**Published:** 2025-01-21

**Authors:** Yuqi Yang, Sen Yue, Luyang Shen, Huiling Dong, Haidong Li, Xiuchao Zhao, Qianni Guo, Xin Zhou

**Affiliations:** ^1^ Key Laboratory of Magnetic Resonance in Biological Systems State Key Laboratory of Magnetic Resonance Spectroscopy and Imaging National Center for Magnetic Resonance in Wuhan Wuhan Institute of Physics and Mathematics Innovation Academy for Precision Measurement Science and Technology Chinese Academy of Sciences–Wuhan National Laboratory for Optoelectronics Huazhong University of Science and Technology Wuhan 430071 China; ^2^ University of Chinese Academy of Sciences Beijing 100049 China

**Keywords:** biosensors, hyperpolarization, magnetic resonance imaging, nanomaterials, the lungs

## Abstract

Magnetic resonance imaging (MRI) is a cornerstone technology in clinical diagnostics and in vivo research, offering unparalleled visualization capabilities. Despite significant advancements in the past century, traditional ^1^H MRI still faces sensitivity limitations that hinder its further development. To overcome this challenge, hyperpolarization methods have been introduced, disrupting the thermal equilibrium of nuclear spins and leading to an increased proportion of hyperpolarized spins, thereby enhancing sensitivity by hundreds to tens of thousands of times. Among these methods, hyperpolarized (HP) ^129^Xe MRI, also known as ultrasensitive ^129^Xe MRI, stands out for achieving the highest polarization enhancement and has recently received clinical approval. It effectively tackles the challenge of weak MRI signals from low proton density in the lungs. HP ^129^Xe MRI is valuable for assessing structural and functional changes in lung physiology during pulmonary disease progression, tracking cells, and detecting target molecules at pico‐molar concentrations. This review summarizes recent developments in HP ^129^Xe MRI, including its physical principles, manufacturing methods, in vivo characteristics, and diverse applications in biomedical, chemical, and material sciences. In addition, it carefully discusses potential technical improvements and future prospects for enhancing its utility in these fields, further establishing HP ^129^Xe MRI's importance in advancing medical imaging and research.

## Graphical abstract

Ultrasensitive ^129^Xe magnetic resonance imaging (MRI) is a novel clinically approved technology that overcomes the sensitivity limitations of traditional ^1^H MRI by introducing hyperpolarized ^129^Xe as the signal source. This review summarizes recent advancements in ^129^Xe MRI, covering its principles, methodologies, applications ranging from clinical assessment to molecular sensing, as well as potential developments that may further enhance its utility.

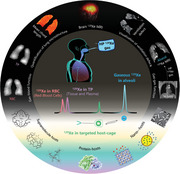

## 1 Introduction

Since Otto Stern's discovery of the proton's magnetic moment in the 1920s, theories, methods, and applications related to magnetic resonance have garnered six Nobel Prizes in physics, chemistry, and physiology or medicine.^[^ ^1^ ^]^ Over the past century, magnetic resonance (MR)‐based technologies have evolved into powerful tools for studying intramolecular and intermolecular interactions,^[^ ^2^ ^]^ physiological structures,^[^ ^3^ ^]^ and biological functions.^[^ ^4^ ^]^ Magnetic resonance imaging (MRI) is widely used in preclinical medicine and clinical diagnosis due to its noninvasive nature, lack of ionizing radiation, and ability to penetrate deep tissues.^[^ ^5^ ^]^ However, its further advancement is hindered by the fact that only approximately one out of every 100 000 atoms contribute to the detection signal in conventional MRI, resulting in sensitivity orders of magnitude lower than that of positron emission tomography (PET), single‐photon emission computed tomography (SPECT), and optical imaging modalities.^[^ ^6^ ^]^

The magnetic resonance signal depends on the nuclei spin polarization, *P*, which characterizes the variation of the nuclei population in different energy states, based on Boltzmann thermal equilibrium polarization (thermal polarization):^[^ ^7^ ^]^

(1) $$P=\frac{N_{\beta }-N_{\alpha }}{N_{\beta }+N\alpha }=\tanh\left(\frac{\mu B_{0}}{k_{B}T}\right)\approx \frac{\mu B_{0}}{k_{B}T}$$

where N_α_ and N_β_ represent the number of spins in the two sublevels, μ is the magnetic moment, B_0_ is the magnetic field, k_B_ is Boltzmann's constant, and T is the sample temperature. Applying higher magnetic fields (*B*_0_) and lowering the temperature (*T*) are commonly used methods to achieve higher polarization. Currently, MRI scanners designed for human use can reach a maximum magnetic field strength of 11.7 T,^[^ ^8^ ^]^ which may theoretically increase polarization by 7.8 times compared to a clinical MRI scanner typically operating at 1.5 T. However, improvements in magnetic field strength necessitate the use of precise and costly hardware. In addition, higher magnetic fields increase the specific absorption rate (SAR), reflecting intensified interactions between the radio frequency (RF) field and biological tissue.^[^ ^9^ ^]^ This can exacerbate image inhomogeneities, complicating the pursuit of high‐quality MRI results and potentially raising clinical safety concerns. Likewise, reducing the temperature is not practical for human clinical examinations, further limiting the enhancement of polarization.

A novel technology, “hyperpolarization,” places the nuclei of interest in contact with a reservoir of highly polarized electrons, disrupting the thermal equilibrium state of Boltzmann distribution and arranging for the high polarization to be transferred to the nuclear spin system of interest.^[^ ^10^ ^]^ Various hyperpolarization methods, including dynamic nuclear polarization (DNP),^[^ ^11^ ^]^ chemical induced dynamics nuclear polarization (CIDNP),^[^ ^12^ ^]^ optical pumping (OP),^[^ ^13^ ^]^ optical nuclear polarization (ONP),^[^ ^14^ ^]^ parahydrogen‐induced polarization (PHIP),^[^ ^15^ ^]^ and quantum rotor induced polarization (QRIP),^[^ ^16^ ^]^ can augment polarization by hundreds to tens of thousands of times and increase the sensitivity by orders of magnitude.

Among these methods, hyperpolarized gas MRI stands out due to its utilization in in vivo studies and its approval for clinical applications. Specifically, ^129^Xe and ^3^He are selected as gaseous agents to study the gas diffusion in lung airspace,^[^ ^6b^ ^]^ because the two atoms could be hyperpolarized and detected by MRI. Given the significant expense, global scarcity and insolubility of ^3^He, ^129^Xe emerges as a more appealing option for extensive clinical use.

An indirect optical pumping technique, known as spin exchange optical pumping (SEOP),^[^ ^17^ ^]^ transfers the angular momentum of photons to the nuclear spin system of interest (^129^Xe) through alkali metal intermediaries. This process disrupts the nuclear spin thermal equilibrium, establishing a new balance wherein at least 1 out of every 10 ^129^Xe atoms can be detected in hyperpolarized (HP) ^129^Xe MRI. Consequently, the nuclear spin polarization, *P*, increases to over 0.1 through hyperpolarization, a significant enhancement compared to the 10^−5^ achieved through thermal polarization. In conventional nuclear magnetic resonance spectroscopy (NMR) and MRI, achieving an acceptable signal intensity for ^129^Xe requires accumulation over thousands of times and acquisition over tens of minutes. However, for hyperpolarized ^129^Xe, a single accumulation in 0.1 s is adequate to yield high signal‐to‐noise results.^[^ ^18^ ^]^ This sensitivity enhancement leads to a substantial reduction in scan time for hyperpolarized gas MRI compared to traditional MRI scans, typically requiring only a few seconds to a dozen seconds to obtain complete sampling.

Due to the significant improvement in sensitivity, hyperpolarized ^129^Xe MRI is often referred to as ultrasensitive ^129^Xe MRI.^[^ ^19^ ^]^ This substantial enhancement in sensitivity has greatly broadened the scope of MRI applications. For instance, while traditional ^1^H MRI has struggled to visualize the lungs due to their low proton density—≈1000 times lower than other tissues—hyperpolarized gas MRI offers a solution. Hyperpolarized (HP) ^129^Xe gas, when inhaled, travels from the windpipe into the lungs and then dissolves in the bloodstream. By tracking the signal from HP ^129^Xe, researchers can map the ventilation function and evaluate gas–blood exchange in the lungs. Consequently, HP ^129^Xe MRI enables visualization of localized ventilation, perfusion, diffusion, and microstructure of the lungs, which are not simultaneously available by other conventional imaging technologies. In addition, the dissolved ^129^Xe atoms can travel to other parts of the body along with the bloodstream, allowing imaging of other organs like the brain and kidneys. Despite its advantages as an MRI signal source, xenon's inertness, which prevents it from reacting with biomolecules, limits its use in molecular imaging. To address this limitation, expandable “hosts” with specific identification components have been introduced to provide suitable space for capturing ^129^Xe atoms and generating a unique magnetic resonance (MR) signal that indicates the state of the guest. By adjusting these components, specific molecules can be targeted, and their presence can be tracked by monitoring the MR signals from guest ^129^Xe. This combination of hyperpolarization and targeting enables the detection of biomolecules at picomolar (pm) levels, a significant improvement over conventional technologies, which typically operate at sub‐millimolar levels.

Given its significantly enhanced sensitivity, specific gas–gas/gas–blood exchange capabilities and broad in vivo applicability, HP ^129^Xe MRI is poised to become a next‐generation imaging technology. Ultrasensitive ^129^Xe MRI offers a pioneering approach with distinct advantages for clinical diagnosis, and it is set to make a profound impact across the fields of medicine, biology, chemistry, and material science. Recently, Chekmenev^[^ ^17^ ^]^ and Schröder^[^ ^20^ ^]^ have provided thorough reviews of the principles, instruments and molecular sensing applications of HP ^129^Xe MRI. This article presents a comprehensive review of the applications of hyperpolarized ^129^Xe magnetic resonance, covering human, animal, cellular and molecular levels while also carefully anticipating future developments that could further enhance its utility.

## 2 The Inhaled ^129^Xe Generates Distinguished Magnetic Signals In Vivo

**Figure 1:**
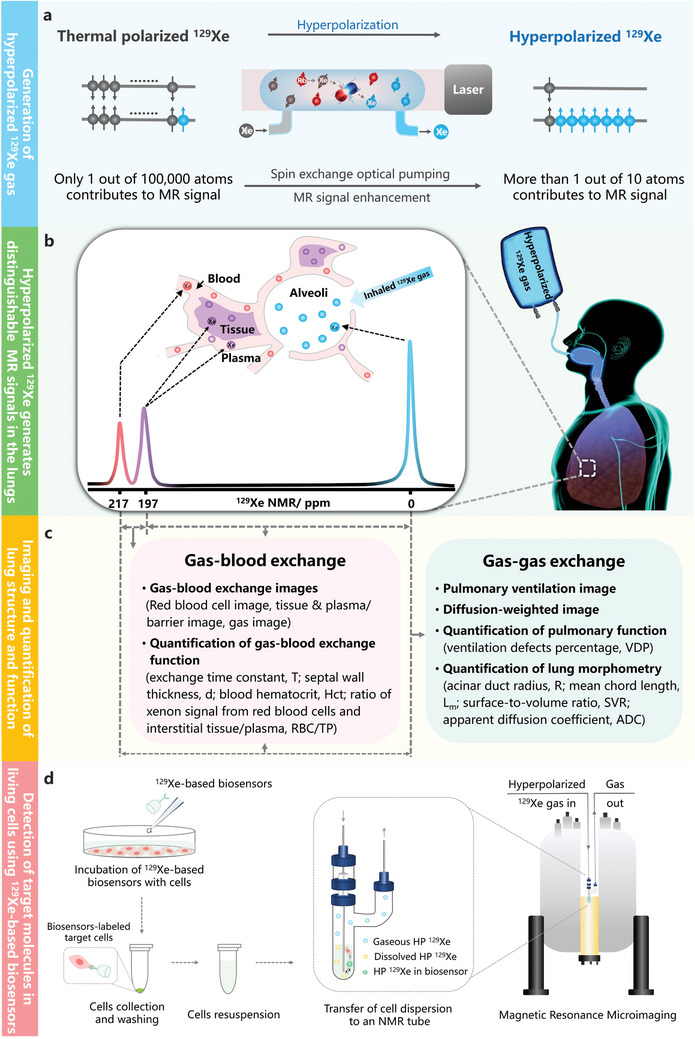
Hyperpolarized (HP) ^129^Xe is introduced to visualize the lungs and monitor target molecules in MRI. a) The process of generating HP ^129^Xe involves the transfer of angular momentum from photons to ^129^Xe atoms via an alkali metal intermediary, leading to an enhancement of the HP ^129^Xe magnetic resonance signal by over 10 000 times compared to that at thermal equilibrium. b) The produced HP ^129^Xe gas is inhaled, generating distinct magnetic resonance signals in pulmonary alveoli (0 ppm), lung tissues and plasma (197 ppm) and red blood cells (217 ppm), respectively. c) Using the diverse signals, ultrasensitive ^129^Xe MRI enables the quantitative assessment of gas–gas and gas–blood exchanges, as well as the visualization of lung structure and function. d) Typical procedures for identifying target molecules within living cells by introducing ^129^Xe‐based biosensors.

To enhance sensitivity, a technique known as spin‐exchange optical pumping (SEOP) is used to hyperpolarize noble gases like ^3^He and ^129^Xe. Chekmenev et al. has extensively discussed the physical processes and instruments of SEOP.^[^ ^17^ ^]^ Briefly, SEOP involves two main steps. Using ^129^Xe as an illustration, the process begins with the excitation and spin‐polarization of the outer‐shell electrons of vaporized alkali metals (e.g., Rubidium) using a circularly polarized laser beam (794.7 nm) in a static magnetic field. Subsequently, the electron polarization of alkali metal atoms is transferred to the nuclear spins of ^129^Xe to produce hyperpolarized gas. As a result of the angular momentum transferred from photons, the polarization of ^129^Xe is enhanced by tens of thousands of times (**Figure** **1**a). According to Goodson et al., a high in‐cell ^129^Xe nuclear spin polarization of over 90% has been achieved under batch mode,^[^ ^21^ ^]^ indicating that up to 90% of the ^129^Xe sources can potentially be detected.

As an inertness gas, xenon can be safely inhaled in clinical settings.^[^ ^22^ ^]^ After polarization, ^129^Xe gas is collected and subsequently inhaled through the respiratory system, followed by 5–10 s breath holding for the following MRI studies. The inhaled ^129^Xe gas traverses the trachea and bronchi, reaches the alveoli, and generates an MR signal at 0 ppm (Figure 1b). This signal enables direct visualization of gas distribution in the lungs. Consequently, regions with strong signals indicate high ^129^Xe gas density and good ventilation function, while weak signals suggest low gas flow and ventilation obstruction (Figure 1c). In contrast to the clinically used pulmonary function tests (PFTs), which evaluate the ventilation function of the entire lung, HP ^129^Xe MRI can visualize localized ventilation defects that may be overlooked due to compensation from healthy areas. Therefore, changes in lung disease progression associated with ventilation (e.g., airway blockage, respiratory stenosis) can be monitored using ultrasensitive ^129^Xe MRI, offering distinct advantages in tracking disease advancement and prognosis.

Using ^129^Xe as a contrast agent offers advantages over other gases due to its lipophilicity, making it soluble in biological barriers. In addition, the remarkable sensitivity of ^129^Xe atoms to their surroundings leads to significant changes in chemical shifts across different environments.^[^ ^23^ ^]^ Consequently, ^129^Xe atoms can permeate the alveolar wall and enter blood and tissues after reaching the alveoli, generating distinct MR signals at 217 and 197 ppm (Figure 1b), respectively. Therefore, in addition to high‐resolution ventilation images, HP ^129^Xe MRI can visualize and quantify gas–gas and gas–blood exchange functions of the lungs (Figure 1c).

The subsequent stage of the dissolved ^129^Xe atom's journey involves either reverting to the gaseous phase within the alveolus before exhalation or being transported via the bloodstream throughout the body. Consequently, perfused organs such as the brain and kidneys could also be imaged using HP ^129^Xe MRI.

The inhaled HP ¹^2^⁹Xe has a T_1_ relaxation time of ≈20 s in the alveoli, 6–8 s in arterial oxygenated blood, and 3–4 s in venous deoxygenated blood.^[^ ^19^, ^24^ ^]^ To ensure adequate signal intensity for MRI, HP ¹^2^⁹Xe MRI sequences typically require scan times on the order of seconds.^[^ ^25^ ^]^ Moreover, HP ¹^2^⁹Xe MRI is typically performed during breath‐holds, necessitating short scan times due to the limited breath‐holding capacity of patients. Clinically, patients with pulmonary diseases generally have lower breath‐hold tolerance compared to healthy individuals. As a result, various accelerated acquisition techniques have been developed for HP ¹^2^⁹Xe MRI to reduce the breath‐hold burden on patients and expand its clinical applications. Therefore, the ultra‐high sensitivity of HP ¹^2^⁹Xe represents a significant advancement over traditional MRI, which often requires several to dozens of minutes for scanning. Specific acceleration methods will be discussed in the following sections.

## 3 Quantification of Lung Microstructure Parameters Using HP ^129^Xe MRI

**Figure 2:**
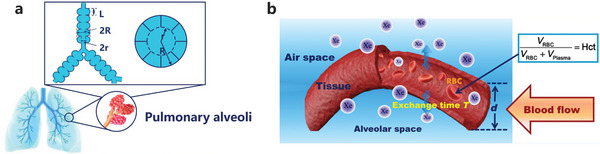
Quantification of lung microstructure and function by using noninvasive HP ^129^Xe MRI. a) The acinar lung airway model that describes the pulmonary microstructure parameters, and b) the diagram of the gas–blood exchange region of the alveoli. Reproduced with permission.^[^ ^26^ ^]^ Copyright 2021, The American Association for the Advancement of Science.

When inhaled into the lungs, the diffusion of ^129^Xe gas is significantly hindered by the alveoli. By analyzing the gas diffusion process, it is possible to quantify certain fundamental geometrical parameters of the acinar airway by examining the relationships between lung microstructure parameters and the diffusion attenuated MR signal. As described by the Weibel model (**Figure** **2**), the alveolar airway is conceptualized as a cylinder comprising a tube embedded in an alveolar sheath. Various geometrical parameters, such as the external radius (*R*), internal radius (*r*), and alveolar length (*L*), are introduced to provide a quantitative description of lung microstructure.

**Figure 3:**
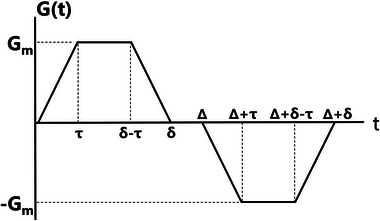
Diffusion sensitive pulse gradient waveform introduced in simulations.

Yablonskiy et al.^[^ ^27^ ^]^ concluded that the diffusion of ^129^Xe gas within each acinar airway is anisotropic and can be considered as diffusing in two directions: along the airway's principal axis, described by longitudinal diffusion coefficient (*D*_L_), and along the transverse plane, described by transverse diffusion coefficient (*D*_T_). Quantifying these parameters is based on a diffusion‐sensitive sequence, the Stejskal–Tanner pulsed field gradient sequence, which utilizes two gradient pulses of opposite polarity (**Figure** **3**) and is characterized by the so‐called *b*‐value:

(2) $$\begin{array}{c} b={\left(\gamma G_{m}\right)}^{2}\cdot \left[\delta^{2}\left(\Delta -\frac{\delta }{3}\right)+\tau \left(\delta^{2}-2\delta \Delta +\Delta \tau -\frac{7}{6}\delta \tau +\frac{8}{15}\tau^{2}\right)\right] \end{array}$$

Here, γ represents gyromagnetic ratio (7.4 G^−1^ · ms^−1^ for ^129^Xe), and *G*_m_ denotes the gradient amplitude. Presently, clinical MRI scanners permit diffusion gradient strengths up to 30–40 mT m^−1^. The optimal diffusion time for ^129^Xe gas in the human lung is Δ = 5 ms. For small animals, the gradient is ten times higher, with an optimal diffusion time of Δ = 1.3 ms.

The morphological parameters of lungs are on the scale of hundreds of micrometers (µm), while the resolution of MRI is several millimeters (mm). Hence, the diffusion‐attenuated signal in each voxel is a sum of signals from individual acinar airways. The total MR signal from a voxel can be described as follows:

(3) $$S\left(b\right)=S_{0}\exp\left(-bD_{T}\right){\left(\frac{\pi }{4bD_{an}}\right)}^{\frac{1}{2}}\cdot \Phi \left[{\left(bD_{an}\right)}^{\frac{1}{2}}\right]$$

(4) $$D_{an}=D_{L}-D_{T}$$

Here, the *b*‐value is determined by the setting of Stejskal–Tanner pulse sequence. *D*_L_ and *D*_T_, which can be confirmed through applying Equations (3) and (4) to multi‐*b*‐value measurements of the ^129^Xe MR signal, are related to lung microstructure and the details of the diffusion‐sensitizing pulse sequence:

(5) $$D_{L}=D_{L0\;}\cdot \left(1-\beta_{L}\cdot bD_{L0}\right)$$

(6) $$D_{T}=D_{T0}\cdot \left(1+\beta_{T}\cdot bD_{T0}\right)$$

For humans, the longitudinal diffusivity:

(7) $$D_{L0}=D_{0}\cdot \exp\left[-2.81\cdot {\left(1-r/R\right)}^{1.76}\right]$$

(8) $$\beta_{L}=21.2\cdot {\left(R/L_{1}\right)}^{3/2}\cdot \exp\left[-3.65\cdot {\left(1-r/R\right)}^{-1/2}\right]$$

and the transverse diffusivity:

(9) $$D_{T0}=D_{0}\cdot \exp\left[-0.74\cdot {\left(L_{2}/R\right)}^{1.47}\right]\cdot \left[1+u\left(r,R\right)\right]$$

(10) $$u\;\left(r,R\right)=c_{1}\left(R\right)\cdot \left(1-r/R\right)+c_{2}\left(R\right)\cdot {\left(1-r/R\right)}^{2}$$

(11) $$c_{1}\;\left(R\right)=1.13\cdot \left(R/L_{2}\right)-1.40\cdot {\left(R/L_{2}\right)}^{2}$$

(12) $$c_{2}\;\left(R\right)=3.55-11.27\cdot \left(R/L_{2}\right)+7.44\cdot {\left(R/L_{2}\right)}^{2}$$

(13)

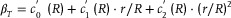



(14)

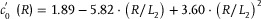



(15)

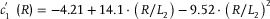



(16)

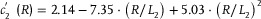

where free diffusion coefficients *D*_0_ is 0.14 cm^2^ s^−1^ for ^129^Xe, *L*_1_ = (2*D*_0_Δ)^1/2^  and *L*_2_ = (4*D*_0_Δ)^1/2^  are the characteristic diffusion length for one‐ and two‐dimensional diffusion, respectively.

Applying Equations (5)–(16) to the MRI signals generated from multi‐*b* MR experiments, the external radius (*R*) and internal radius (*r*) of alveolar could be carefully determined. Other lung microstructure related physiological parameters (surface‐to‐volume ratio *S*/*V*, mean chord length *L*_m_, alveolar density *N*_a_) are further calculated:

(17) $$S/V=\frac{2\pi R\cdot L+2\pi \cdot \left(R^{2}-r^{2}\right)+16\left(R-r\right)\cdot L}{\pi R^{2}L}$$

(18) $$L=2Rsin\;\frac{\pi }{8}=0.765R$$

(19) $$L_{m}=4\cdot V/S,N_{a}=1/\left(\pi R^{2}L\right)$$

In mice and rats, the geometrical parameters of acinar airway and the optimal diffusion time Δ are much smaller than that of in human lungs. Consequently, the equations describing the longitudinal and transverse diffusion coefficients need to be adjusted as follows:

(20) $$D_{L0}=D_{0}\cdot \exp\left[-2.89\cdot {\left(1-r/R\right)}^{1.78}\right]$$

(21) $$\beta_{L}=35.6\cdot {\left(R/L_{1}\right)}^{3/2\;}\cdot \exp\left[-4\cdot {\left(1-r/R\right)}^{-1/2}\right]$$

(22) $$D_{T0}=D_{0}\cdot \exp\left[-0.73\cdot {\left(L_{2}/R\right)}^{1.4}\right]\cdot \left[1+u\left(r,R\right)\right]$$

(23) $$\begin{array}{ccc} u\;\left(r,R\right) & = & \exp\left[-A\left(R\right)\cdot {\left(1-r/R\right)}^{2}\right]\cdot \left[exp(-5\cdot {\left(1-r/R\right)}^{2}\right)\\ & & +\;5\cdot {\left(1-r/R\right)}^{2}-1] \end{array}$$

(24) $$A\;\left(R\right)=1.3+0.25\cdot \exp\left[14\cdot {\left(R/L_{2}\right)}^{2}\right],\beta_{T}=0.06$$

The results from actual samples show that the lung geometrical parameters obtained from HP ^129^Xe MRI are highly consistent with those from pathological sections.^[^ ^28^ ^]^ Despite pathological sections having been the gold standard for evaluating lung microstructures for decades, their application is still limited due to the invasiveness, complicated staining procedures, and long waiting times. HP ^129^Xe MRI provides a noninvasive tool for continuously monitoring lung structure at the alveolar level, capable of quantifying lung microstructure parameters in a single breath hold (≤16 s).^[^ ^25^ ^]^ Thus, HP ^129^Xe MRI offers a novel approach to identifying changes in lung geometrical parameters during disease progression, applicable not only to major pulmonary diseases such as lung cancer but also to chronic lung diseases related to structure, such as pulmonary fibrosis and emphysema.

Another alternative theoretical model for quantifying the diffusion behavior in the lung is the stretched exponential model (SEM).^[^ ^29^ ^]^ This model estimates signal attenuation related to diffusion through the probability density function or diffusion propagator, with the mean diffusion length (Lm_D_) providing information about alveoli size.

The relationship between *L*_m_ Equation (19) and *L*m_D_ can be described as follows:^[^ ^30^ ^]^

(25) $$L_{m}=-562\;\mu m+4.3\cdot Lm_{D}\cdot \sqrt{\frac{2D_{0}^{He}\Delta_{He}}{2D_{0}^{Xe}\Delta_{Xe}}}$$

where $D_{0}^{He}$ is the free diffusion coefficient of ^3^He, Δ_He_ = 1.46 ms, $D_{0}^{Xe}$ is the free diffusion coefficient of ^129^Xe, and Δ_Xe_ is the diffusion time in ^129^Xe DWI measurements.

After diffusing in the alveoli, ^129^Xe travels through lung tissue and plasma before eventually binding with red blood cells (RBCs). This process can be described as a model that allows for the calculation of physiological parameters related to gas–blood exchange. Initially, Mansson et al.^[^ ^31^ ^]^ proposed a gas–blood exchange model in which the alveoli were assumed to have a circularly symmetric geometry, with capillaries surrounding their outer surface. The model described the diffusion process of ^129^Xe from the alveoli into the blood and obtained membrane diffusing capacity and pulmonary perfusion. Later, new models^[^ ^32^ ^]^ were developed that considered ^129^Xe diffusion along one direction, with blood flow perpendicular to this diffusion. In these models, the signals from the tissue and RBC compartments were not distinguished; instead, they were treated as a single signal within the septum and analyzed using a one‐dimensional diffusion equation. Furthermore, the model of xenon exchange (MOXE)^[^ ^33^ ^]^ was established that considered dissolved ^129^Xe in both tissue (S_d1_, 197 ppm) and blood (S_d2_) separately. In addition, the blood signal was divided into contributions from plasma (197 ppm) and RBCs (217 ppm) by using the fraction of xenon in RBCs relative to the total xenon in the blood (η) (Figure 2b). Thus, the model accounted for three components of the septum—tissue, plasma, and RBCs—and allowed the signals from these components to be correlated with the TP and RBC signals observed in experiments using the following equations:

(26) $$S_{TP}\;\left(t\right)=S_{d1}\;\left(t\right)+\left(1-\eta \right)S_{d2}\left(t\right)$$

(27) $$S_{RBC}\;\left(t\right)=\eta \cdot S_{d2}\left(t\right)$$

By solving the one‐dimensional diffusion equation, the distribution of dissolved ^129^Xe can be obtained:

(28) $$M_{d}\;\left(x,t\right)=\lambda M_{f}\;\left\{1-\frac{4}{\pi }\sum_{n=odd}\frac{1}{n}\sin\frac{n\pi x}{d}e^{-n^{2}t/T}\right\}$$

where *M*_d_ and *M*_f_ represent the density of dissolved and gaseous ^129^Xe in the lung, respectively. λ represents the Ostwald solubility of xenon in lung parenchyma, and *T* is the xenon‐exchange time constant in the lung, $T=\frac{d^{2}}{\pi^{2}D}\;$, *D* is the diffusion coefficient of dissolved xenon. To quantify the signal intensity, the normalized signal distribution of dissolved ^129^Xe in the lung relative to gaseous ^129^Xe was obtained from Equation (29),

(29) $$S_{d}\;\left(x,t\right)=\frac{\lambda }{2}\;\frac{S_{A}}{V_{g}}\left\{1-\frac{4}{\pi }\sum_{n=odd}\frac{1}{n}\sin\frac{n\pi x}{d}e^{-n^{2}t/T}\right\}$$

where *S*_A_ and *V*_g_ are the total surface area and total volume of the air spaces in the lung, respectively. Considering the effect of blood flow, calculating *S*_d1_ and *S*_d2_ individually from Equation (29) and combining Equations (26) and (27), the temporal signal distribution of the dissolved ^129^Xe in TP and RBC can be calculated as follows:

(30) $$\begin{array}{ccc} & & S_{\mathrm{TP}}\;\left(t\right)=b\left[\frac{2\delta }{d}-\frac{8}{\pi^{2}}\sum_{n=\mathrm{odd}}\frac{1}{n^{2}}\left(1-\cos\frac{n\pi \delta }{d}\right)e^{-\frac{n^{2}t}{T}}\right]\\ & & \;+\;b\left(1-\eta \right)\left\{2\left[\left(1-\frac{2\delta }{d}\right)\frac{t}{t_{X}}-\frac{8T}{\pi^{2}t_{X}}\sum_{n=\mathrm{odd}}\frac{1}{n^{4}}\cos\left(\frac{n\pi \delta }{d}\right)\left(1-e^{-n^{2}t/T}\right)\right]\right.\\ & & \;\left.+\;\left(1-\frac{t}{t_{X}}\right)\left[\left(1-\frac{2\delta }{d}\right)-\frac{8}{\pi^{2}}\sum_{n=\mathrm{odd}}\frac{1}{n^{2}}\cos\left(\frac{n\pi \delta }{d}\right)e^{-n^{2}t/T}\right]\right\} \end{array}$$

(31) $$\begin{array}{ccc} & & S_{\mathrm{RBC}}\;\left(t\right)=b\eta \left\{2\left[\left(1-\frac{2\delta }{d}\right)\frac{t}{t_{X}}-\frac{8T}{\pi^{2}t_{X}}\sum_{n=\mathrm{odd}}\frac{1}{n^{4}}\cos\left(\frac{n\pi \delta }{d}\right)\left(1-e^{-n^{2}t/T}\right)\right]\right.\\ & & \;\left.+\;\left(1-\frac{t}{t_{X}}\right)\left[\left(1-\frac{2\delta }{d}\right)-\frac{8}{\pi^{2}}\sum_{n=\mathrm{odd}}\frac{1}{n^{2}}\cos\left(\frac{n\pi \delta }{d}\right)e^{-n^{2}t/T}\right]\right\} \end{array}$$

where $b=\frac{\lambda d}{2}\;\frac{S_{A}}{V_{g}}$ is the normalization factor (dimensionless), δ/*d* is the barrier‐to‐septum ratio, and *t*_x_ is the pulmonary capillary transit time. By fitting the signals acquired through chemical shift saturation recovery (CSSR) with these formulas, physiological parameters can be obtained. Hematocrit (Hct) can be calculated from the η parameter:

(32) $$Hct=\frac{V_{RBC}}{V_{RBC}+V_{Plasma}}=\frac{\eta /\lambda_{RBC}}{\eta /\lambda_{RBC}+\left(1-\eta \right)/\lambda_{P}}\;$$

where λ_RBC_ and λ_P_ are Ostwald solubilities of xenon in RBCs and plasma, respectively.

## 4 Applications of HP ^129^Xe MRI for Lung Disease

Pulmonary diseases loom as a formidable global health challenge. According to World Health Organization (WHO), five out of the ten leading causes of deaths in 2021 were pulmonary diseases,^[^ ^34^ ^]^ including coronavirus disease (COVID‐19), chronic obstructive pulmonary disease (COPD), lower respiratory infections, trachea, bronchus, lung cancers, and tuberculosis. With symptoms ranging from mild discomfort to life‐threatening complications, pulmonary diseases not only diminish quality of life but also contribute to increased mortality rates.^[^ ^35^ ^]^ Tackling this issue demands concerted efforts in prevention, diagnosis, treatment, and research on a global scale.

Imaging techniques such as computed tomography (CT), positron emission tomography (PET), and magnetic resonance imaging (MRI) provide direct insight into evaluating structural or functional changes in tissues or organs during disease progression. Although CT and extended CT methods are able to effectively evaluate pulmonary structure with high spatial resolution^[^ ^36^ ^]^ and derive the ventilation function of lung,^[^ ^37^ ^]^ respectively, they cannot assess the gas exchange function and is unsuitable for repeated use over a short period of time due to exposure to ionizing radiation. PET offers dynamic metabolism information, particularly valuable for lung cancer diagnosis.^[^ ^38^ ^]^ However, the existing factors such as radioactivity, high price, and poor spatial resolution restrict its application in the diagnosis of common lung diseases. MRI is a frequently used imaging technique in clinical settings, primarily for soft tissue imaging.^[^ ^39^ ^]^ Clinical MRI is advantageous as it does not involve ionizing radiation and is suitable for long‐term monitoring. Nevertheless, the inherently low proton density in lung tissue results in poor MRI signal quality, hindering its effectiveness in pulmonary imaging.

**Figure 4:**
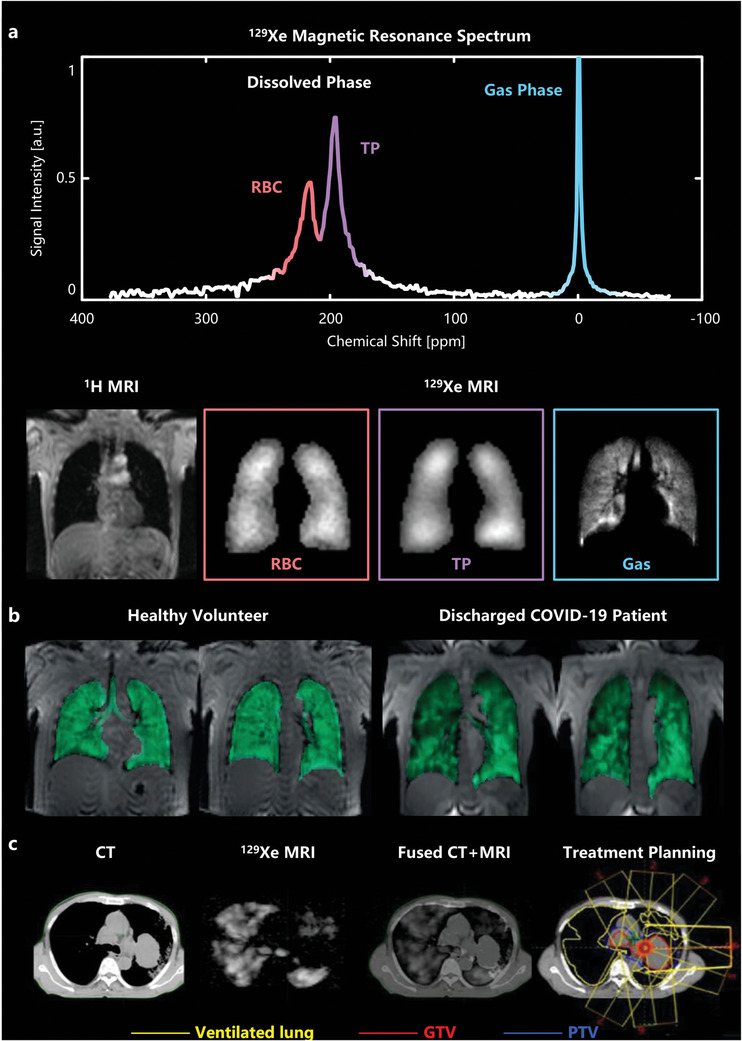
Images of human chest. a) The ^1^H MRI reveals a “black hole” in the lungs due to the lack of signal sources from protons. The HP ^129^Xe magnetic resonance spectrum and images show that ^129^Xe in the lung airspace registers a signal at 0 ppm, while dissolved ^129^Xe in TP and RBC shows signals around 197 and 217 ppm, respectively. b) A comparison of ventilation images between a healthy volunteer and a discharged COVID‐19 patient. Reproduced with permission.^[^ ^44^ ^]^ Copyright 2024, Wiley. c) ^129^Xe MRI provides functional information of the lungs to guide radiotherapy planning. Reproduced with permission.^[^ ^45^ ^]^ Copyright 2022, Wiley Periodicals, LLC on behalf of The American Association of Physicists in Medicine.

Fortunately, gas contrast agents can facilitate density‐weighted imaging in the lungs, and noble gases emerge as suitable options due to their safety and minimal side effects on humans. In addition, the application of hyperpolarization techniques, such as spin exchange optical pumping,^[^ ^40^ ^]^ enables the enhancement of the MR signal of noble gas by more than 10 000 times, referred to “hyperpolarized (HP) gas,” thereby enabling lung gas MRI. Helium‐3 (^3^He) and xenon‐129 (^129^Xe) are the commonly utilized noble gases for lung MRI owing to their relatively long longitudinal relaxation time. In comparison with ^3^He, HP ^129^Xe gas offers unique advantages for lung MRI: 1) ^129^Xe is an atmospheric gas with a more abundant supply on Earth and is less expensive; 2) ^129^Xe gas exhibits strong lipophilicity, allowing it to dissolve into the blood, whereas ^3^He is virtually insoluble in any substance;^[^ ^41^ ^]^ 3) xenon is sensitive to its local chemical environment, manifesting distinct chemical shifts in alveoli, TP and RBC, denoted as 0, 197, and 217 ppm, respectively (Figure 1b).^[^ ^42^ ^]^ These shifts can be utilized to evaluate pulmonary gas exchange function (**Figure** **4**a). Furthermore, the safety and tolerability of HP ^129^Xe MRI have been validated not only in healthy adults but also in children and patients with lung diseases, without any severe adverse events.^[^ ^43^ ^]^ Therefore, HP ^129^Xe MRI holds tremendous potential for lung imaging and disease assessment.

### 4.1 Pulmonary Ventilation Function Assessment

As an exogenous signal source for MRI, ^129^Xe has no background noise in tissues. Consequently, HP ^129^Xe MRI could directly visualize gas distribution in the alveoli, indicating the local ventilation in the lungs. In healthy subjects, ^129^Xe gas would flow smoothly and distribute homogeneously throughout the lungs. In contrast, patients with impaired lung function display inhomogeneous ^129^Xe distribution and various ventilation defects on the gas images due to the airway obstruction and/or the destruction of lung parenchyma.

To quantify ventilation function, a parameter named ventilation defect percentage (VDP) is proposed. VDP is defined as the ratio of the areas with no or hypointense signal in ^129^Xe images to the total lung volume, as determined by proton MR images. The feasibility of VDP used for evaluating lung diseases has been demonstrated in numerous studies, with higher VDP values observed in patient with COVID‐19,^[^ ^26^, ^44^, ^46^ ^]^ COPD,^[^ ^47^ ^]^ asthma,^[^ ^48^ ^]^ cystic fibrosis (CF),^[^ ^49^ ^]^ and lymphangioleiomyomatosis^[^ ^50^ ^]^ compared with healthy volunteers (**Table** **1**). In addition, previous studies have confirmed a strong correlation of VDP with clinical lung disease evaluation index, such as forced expiratory volume in the first second (FEV_1_)^[^ ^47^, ^48^, ^49^ ^]^ and lung clearance index (LCI),^[^ ^49^, ^51^ ^]^ supporting the reliability of ^129^Xe MRI. VDP has also been used to investigate treatment response^[^ ^52^ ^]^ for longitudinal assessment,^[^ ^46^, ^53^ ^]^ showing potential as a clinical endpoint. In the delineation of disease severity or the description of disease pathology, ^129^Xe MRI may emerge as an important diagnostic criteria due to its sensitivity in identifying lung function.^[^ ^48^, ^54^ ^]^

**Table 1:** The gas–gas exchange function, gas–blood exchange function, and lung morphometry parameters quantitatively evaluated by HP ^129^Xe MRI for lung diseases. AATD, alpha‐1 antitrypsin deficiency; CF, cystic fibrosis; COPD, chronic obstructive pulmonary disease; COVID‐19, coronavirus disease 2019; IPF, idiopathic pulmonary fibrosis; LAM, lymphangioleiomyomatosis; NSIP, nonspecific interstitial pneumonia. ADC, apparent diffusion coefficient; *d*, septal wall thickness; δ, barrier thickness; Hct, blood hematocrit; *L*_m_, mean airspace chord length; RBC, red blood cells; TP, Tissue/plasma; RBC/TP, ratio of xenon signal from red blood cells and interstitial tissue/plasma; VDP, ventilation defects percent.

	Gas–gas exchange function			Gas–blood exchange function			
	Ventilation	Lung morphometry parameters		*d* [µm]	δ [µm]	Hct	RBC/TP
	VDP [%]	*L*_m_ [µm]	ADC [cm^2^ s^−1^]				
Health^[^ ^47b^ ^]^	1.3 ± 1.0	211 ± 21	0.034 ± 0.003	9.9 ± 0.7	1.0 ± 0.3	0.22 ± 0.04	0.43 ± 0.11
COPD^[^ ^47^, ^65^, ^93^ ^]^	59.4 ± 9.14	422 ± 174	0.051 ± 0.013	12.1 ± 0.7	1.5 ± 0.4	0.14 ± 0.03	0.23 ± 0.05
AATD^[^ ^67a^ ^]^	–	620 ± 80	0.08 ± 0.01	–	–	–	–
IPF^[^ ^65^, ^77^, ^92^ ^]^	34.1 ± 6.44	365 ± 157	0.046 ± 0.011	17.2 ± 1.1	–	0.15 ± 0.01	0.13 ± 0.04
CF^[^ ^69^, ^93^ ^]^	39.1 ± 13.86	176 ± 41	0.034 ± 0.005	–	–	–	–
NSIP^[^ ^115^ ^]^	4.4 (1.5, 8.7)	–	–	–	–	–	0.24 (0.19, 0.31)
COVID‐19^[^ ^26^ ^]^	5.9 ± 2.9	210 ± 13	0.033 ± 0.003	11.7 ± 2.1	–	0.211 ± 0.048	0.279 ± 0.043
Asthma^[^ ^52^, ^79^ ^]^	17 ± 10	–	–	–	–	–	0.31 ± 0.12
LAM^[^ ^50^ ^]^	19.2(14.8–23.5)	–	0.048(0.042–0.053)	–	–	–	–
Asymptomatic smokers^[^ ^47^, ^68^ ^]^	4.3 ± 1.7	267 ± 39	0.0454 ± 0.0058	10.9 ± 1.8	1.4 ± 0.5	0.16 ± 0.04	0.27 ± 0.07

Since static ¹^2^⁹Xe ventilation images were generally scanned under breath‐hold condition, researchers have implemented various optimizations in trajectory design and reconstruction methods to achieve more efficient acquisition. By utilizing non‐Cartesian sampling trajectories—such as radial,^[^ ^55^ ^]^ spiral,^[^ ^56^ ^]^ and zigzag trajectories^[^ ^44^ ^]^—improvements in ventilation image resolution or reductions in acquisition time can be realized. Compressed sensing (CS), which capitalizes on the inherent sparsity of MR images,^[^ ^57^ ^]^ along with deep learning, which is capable of learning complex patterns and features from existing data,^[^ ^58^ ^]^ facilitates the reconstruction of images even under highly sampled acquisition conditions. This results in image quality comparable to that of fully sampled images, significantly reducing the acquisition time and alleviating the breath‐hold burden for patients.

During the respiratory process, measuring the flow of gases and the expansion of the lungs are also important. Therefore, in addition to static ventilation images, incorporating the temporal information into HP ^129^Xe MRI may enhance the differentiation of disease manifestations. Just as static ventilation images benefit from acceleration techniques, high spatial‐temporal resolution dynamic ventilation images could also be acquired using high‐efficiency trajectories, such as spiral,^[^ ^59^ ^]^ or combining CS technique^[^ ^60^ ^]^ into reconstruction, which allows for the acquisition of 33 frames within 6.67 s.^[^ ^60b^ ^]^ These dynamic images are capable of monitoring ventilation throughout the entire breathing cycle. For instance, the signal–time curves showed statistically significant differences between COPD patients and healthy subjects, revealing delayed ventilation in specific lung regions of interest (ROI).^[^ ^59^ ^] 129^Xe multi‐breath washout (MBW) imaging is another solution that can help identify slow ventilation regionals or areas having more stable ventilation dynamics over the time with fractional ventilation map.^[^ ^61^ ^]^ This technique has been applied in CF volunteers,^[^ ^62^ ^]^ which may be more suitable for the assessment of regional ventilation heterogeneity.

### 4.2 Pulmonary Microstructure Assessment

Some lung diseases are characterized by the damage, weakness or even rupture and fusion of alveoli, including conditions such as emphysema.^[^ ^63^ ^]^ These changes result in a reduced surface area of the lungs, adversely affecting gas–blood exchange. By leveraging the restricted diffusion properties of gas within the lung, ^129^Xe diffusion‐weighted‐imaging (DWI) offers unique advantages for sensitive detection of lung morphological changes, as detailed in Part 3. This technique quantitatively assesses lung microstructures by applying diffusion‐sensitizing gradients (Figure 3). The derived apparent diffusion coefficient (ADC), a commonly used parameter for evaluating gas diffusion capacity, is particularly sensitive to enlarged airspaces.^[^ ^28^, ^64^ ^]^ When combined with theoretical models, multi‐*b*‐values DWI can also provide pulmonary microstructure parameters (as detailed discussed in Part 3), such as external airway radii (*R*), mean chord length (*L*_m_), or mean diffusion length scale (*L*_mD_), allowing for direct quantification of pulmonary morphology.^[^ ^27^, ^65^ ^]^

Notably, subjects with chronic obstructive pulmonary disease (COPD),^[^ ^64^, ^66^ ^]^ alpha‐1 antitrypsin deficiency (AATD),^[^ ^30^, ^67^ ^]^ and idiopathic pulmonary fibrosis (IPF)^[^ ^28^, ^65^ ^]^ exhibit significantly higher ADC and larger *L*_m_ or *L*_mD_ compared with healthy volunteers (Table 1), demonstrating that ^129^Xe DWI could effectively assess the enlargement or destruction of alveoli. Comparisons with pulmonary function tests (PFTs) results reveal moderate but significant correlations between ADC and FEV_1_, FEV_1_/FVC and DL_CO_.^[^ ^50^, ^64^, ^68^ ^]^ In addition, the distribution gradient of ADC might also be altered by the influence of disease. In patients with COPD, ADC displayed a greater superior–inferior reduction consistent with lung tissue destruction, and the anterior‐posterior reduction observed in healthy volunteers caused by tissue compression from gravity was not evident in COPD patients.^[^ ^66a^ ^]^ In addition, smoking, a known major contributor to COPD, can cause lung damage, and similar morphological patterns have been observed in smokers even without pronounced pathological signs.^[^ ^65^, ^68^ ^]^ Furthermore, age is another factor that leads to microstructural lung changes, as evidenced by increased ADC in older healthy volunteers.^[^ ^66^, ^69^ ^]^

Although ^129^Xe DWI has proven to be a powerful tool for exploring lung microstructures, the long acquisition times required for multiple *b*‐value DWI pose a significant challenge due to the need for diffusion gradients and the use of multi‐*b*‐value sequences.^[^ ^25^ ^]^ This underscores the importance of developing rapid acquisition techniques to overcome this limitation. CS is currently the most widely used method for accelerating DW‐MRI acquisition,^[^ ^30^, ^66^, ^67^, ^68^, ^70^ ^]^ achieving high acceleration factors while maintaining image quality. Deep learning has also been effective in reconstructing undesampled DW‐MRI data.^[^ ^71^ ^]^ In addition, rapid, isotropic and whole‐lung covered ^129^Xe DWI has been implement using spiral sequences,^[^ ^72^ ^]^ with the feasibility demonstrated by the ADC results.

### 4.3 Pulmonary Gas–Blood Exchange Function Assessment

The exchange of gas and blood in the lungs represents the initial phase of oxygen entering the body's circulatory system, which is vital for sustaining the body's overall physiological functions. In brief, oxygen inhaled into the alveoli transfers through lung tissue into the bloodstream, where it binds to RBCs before being transported to various organs and tissues. Given that ^129^Xe dissolves in the bloodstream in a manner like oxygen and generates distinct signals from tissue and plasma (TP) and RBC due to the different chemical environments, HP ^129^Xe MRI has emerged as a promising technique for evaluating gas–blood exchange function.

Inflammation, fibrosis, and some lung‐related diseases may impede the gas–blood exchange process. IPF is a prime example of such a condition, characterized by the thickening and fibrosis of alveolar.^[^ ^73^ ^]^ In ^129^Xe gas‐transfer spectra,^[^ ^74^ ^]^ a decrease in RBC signal and an increase in barrier signal were observed in IPF group, indicating that the thickened barrier impeded ^129^Xe delivery and restricted the gas‐exchange function. The reduced ratio of xenon signal from RBCs and TP (RBC/TP) correlated well with lung diffusing capacity for carbon monoxide (DL_CO_), highlighting its potential for assessing the diffusion ability of the alveolar capillary membrane. The sensitivity of HP ^129^Xe spectroscopy was further explored in the longitudinal assessment of IPF patients.^[^ ^75^ ^]^ A significant reduction in RBC/TP was observed after 12 months, with no change in DL_CO_, demonstrating that RBC/TP might be a more sensitive marker for short‐term disease progression.

CSSR is a commonly used technique to estimate dynamic information of dissolved ^129^Xe signal in the lungs.^[^ ^25^, ^76^ ^]^ When combined with the kinetics of HP ^129^Xe across the alveoli, CSSR enables for the quantification of lung function and structure using theoretical model of gas exchange (as detailed discussed in Part 3).^[^ ^31^, ^32^, ^33^ ^]^ These approaches provides precise parameter values, including exchange time constant (*T*), septal wall thickness (*d*), and RBC/TP. Compared to healthy volunteers, the CSSR curves in IPF patients revealed a longer ^129^Xe uptake time and reduced RBC/TP, indicating delayed and impaired gas–exchange function. In addition, the septal wall thickness was significantly different between the two groups, directly reflecting the lesions in IPF patients.^[^ ^77^ ^]^ CSSR has also been applied in COVID‐19^[^ ^26^, ^78^ ^]^ and COPD^[^ ^79^ ^]^ patients, as well as various animal models, including those with emphysema,^[^ ^80^ ^]^ acute lung injury,^[^ ^81^ ^]^ radiation‐induced^[^ ^82^ ^]^ and PM_2.5_‐caused^[^ ^83^ ^]^ lung injury, demonstrating abnormalities in gas‐exchange function.

DWI and CSSR can be used to evaluate the diffusion information within the alveoli and the gas–blood exchange information across the membrane, respectively. Combination of the two techniques within a single breath‐hold was development^[^ ^84^ ^]^ to simultaneously acquire comprehensive microstructural and functional information about both the alveoli and the membrane. This approach avoids the variability caused by different lung inflation level on physiological parameters^[^ ^85^ ^]^ and makes more efficient use of the magnetization of polarized gas, reducing both the burden of breath‐hold on the patient and the cost of the gas.

Since ^129^Xe spectroscopy only provides global information, it is necessary to introduce imaging techniques to characterize the spatial heterogeneity of pathological function damage in the lungs. However, there are three main challenges in imaging dissolved ^129^Xe. First, the concentration of dissolved ^129^Xe is relatively low compared to gaseous ^129^Xe in the alveolar airspace, with the dissolved signal accounting for only about 2% of the total lung ^129^Xe signal.^[^ ^74^ ^]^ Second, the T_2_^*^ of dissolved ^129^Xe is very short because of the inhomogeneous local magnetic field in lung—≈2 ms at 1.5T^[^ ^86^ ^]^—necessitating a short echo time (TE) during scanning. Third, distinguishing ^129^Xe signals from TP and RBC is challenging due to their relatively close chemical shifts.

Initially, researchers directly imaged the signals in both gas and dissolved phase^[^ ^87^ ^]^ without distinguishing signals between TP and RBC, observing different image manifestation of gas uptake and ventilation in patients. Later, techniques were developed to separately image ^129^Xe signals in TP and RBC, including direct imaging,^[^ ^88^ ^]^ interleaved 3D radial 1‐point Dixon acquisition,^[^ ^89^ ^]^ four‐echo 3D radial spectroscopic imaging,^[^ ^90^ ^]^ IDEAL,^[^ ^91^ ^]^ and 3D single‐breath chemical shift imaging.^[^ ^92^ ^]^ These methods provide separate ^129^Xe images in alveoli, TP and RBC, enabling the creation of RBC/TP, RBC/gas, and TP/gas maps that locally characterize pulmonary gas–blood exchange function. These techniques have been utilized to assess pulmonary function changes in patients with asthma,^[^ ^79a^ ^]^ COPD,^[^ ^79^, ^93^ ^]^ left heart failure (LHF),^[^ ^93a^ ^]^ pulmonary arterial hypertension (PAH),^[^ ^93a^ ^]^ COVID‐19,^[^ ^94^ ^]^ CF,^[^ ^93c^ ^]^ and IPF.^[^ ^89^, ^90^, ^92^, ^93^, ^95^ ^]^ Results have shown reduced RBC/TP values, indicating gas–blood transfer restrictions in these patients’ lungs. Furthermore, RBC/TP maps were demonstrated to have significant correlations with DL_CO_, and the abnormal areas on these maps correlated well with the fibrotic areas seen in HRCT images.^[^ ^92^ ^]^

In addition, dynamic imaging of ^129^Xe signals in alveoli, TP and RBC can better characterize the local gas transfer kinetics. This has been studied in healthy volunteers and emphysema patients through IDEAL time‐series images, capturing the complete transfer of ^129^Xe from the alveoli into TP and RBC, and then into the pulmonary vasculature and the left ventricle of the heart.^[^ ^96^ ^]^ Moreover, combining dynamic dissolved‐phase images with the model of xenon exchange can provide more detailed, localized information about gas–blood exchange.^[^ ^97^ ^]^ Due to the higher acceleration require for scan times, further optimization is necessitated in the future.

Pulmonary circulation is a crucial component of gas exchange and exhibits periodic, cardiogenic oscillations, which can be quantified using ^129^Xe dynamic spectroscopy.^[^ ^93^, ^98^ ^]^ Results showed larger oscillations in amplitude, chemical shift and the phase of ^129^Xe signal in RBC in IPF cohort, potentially due to a reduced number of capillaries and delayed oxygenation. In maps of the oscillation amplitudes,^[^ ^99^ ^]^ higher RBC amplitude oscillation was observed in IPF group and lower oscillation was in patients with PAH, which may provide new insights into lung microvasculature. Furthermore, the oscillation of ^129^Xe signals could be used to measure the pulmonary hematocrit in the blood,^[^ ^100^ ^]^ aiding in the detection of hematocrit changes caused by pulmonary diseases.

### 4.4 HP ^129^Xe MRI Guided Therapy

Beyond disease detection, ^129^Xe images also hold promise for guiding lung surgery and radiation therapy (RT), as the areas of ventilation or gas–blood exchange obstruction can be directly visualized. In 2020, Hall et. al. utilized HP ^129^Xe MRI to select the most damaged airways for guiding bronchial thermoplasty (BT).^[^ ^101^ ^]^ In RT, the function map generated by HP ^129^Xe MRI could facilitate function‐dose‐based radiation planning, helping to protect lung areas with preserved function from radiation exposure.^[^ ^45^, ^102^ ^]^

### 4.5 Beyond Lung MRI

Once dissolved into the blood, ^129^Xe is transported via systemic circulation flow to other organs. Owing to its high lipid solubility, ^129^Xe can cross the blood–brain barrier and dissolve in various brain tissues. The complex chemical environment in brain results in five distinguishable peaks in ^129^Xe magnetic resonance spectroscopy,^[^ ^103^ ^]^ corresponding to soft muscular tissue (188 ppm), white matter/ cartilaginous soft‐tissue (192 ppm), gray matter (196 ppm), fluid/plasma/fat tissue outside the brain/ cerebrospinal fluid (200 ppm), and RBC (217 ppm) in human brain. This suggests that ^129^Xe may aid in quantitatively characterizing brain changes and revealing pathologies. To better understand the transfer process of ^129^Xe in the brain, Rao et. al. developed a tracer kinetic model of xenon uptake.^[^ ^104^ ^]^ In addition to spectroscopy, ^129^Xe brain images have been obtained using chemical shift imaging,^[^ ^103^, ^105^ ^]^ gradient echo,^[^ ^106^ ^]^ and time of flight^[^ ^107^ ^]^ pulse sequence, providing information on regional xenon uptake or brain perfusion. Previous studies have demonstrated that ^129^Xe signal in brain is sensitive to blood oxygen content^[^ ^108^ ^]^ and cerebral blood perfusion,^[^ ^109^ ^]^ potentially provide significant complementary information comparable to ^1^H blood oxygenation level‐dependent (BOLD) MRI.^[^ ^107^, ^110^ ^]^

Compared to conventional gadolinium‐based contrast agents, ^129^Xe is safer and does not accumulate in the brain or kidney, making it more suitable for use in patients. Differences in ^129^Xe brain uptake images or wash‐out parameters have been observed in stroke^[^ ^109^, ^111^ ^]^ and Alzheimer's disease,^[^ ^112^ ^]^ indicating potential application for assessing brain diseases by HP ^129^Xe MRI.

Beyond the brain, HP ^129^Xe MRI has also been applied to kidneys^[^ ^113^ ^]^ and brown adipose tissue (BAT).^[^ ^114^ ^]^ Abdomen spectroscopy reveals peaks from the aorta and kidneys, and dynamic images can track the transfer process of dissolved ^129^Xe to the kidneys. Moreover, the increased ^129^Xe uptake in BAT during the stimulation in mice has been observed, demonstrating the potential of HP ^129^Xe MRI for measuring energy metabolism.

## 5 Functionalized ^129^Xe Biosensors for Molecular Imaging

**Figure 5:**
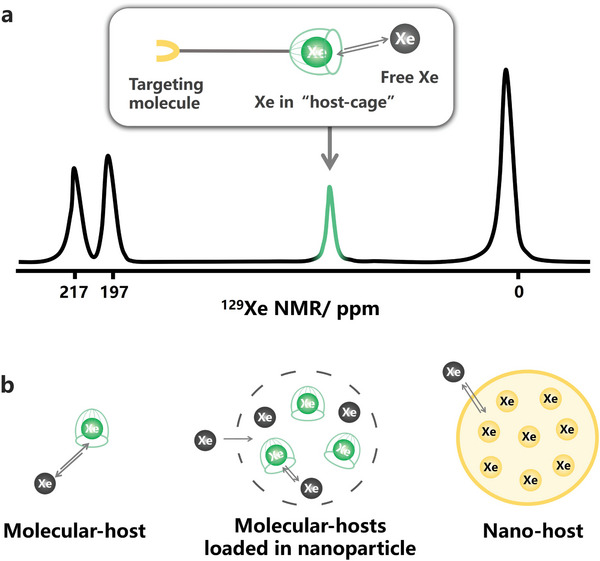
Endowing targeting ability to inert ^129^Xe. a) In a typical ^129^Xe probe, the host‐cage identifies targets by the guidance of the modified targeting molecule, and then captures ^129^Xe atoms to produce a specific MR signal. b) The exchange process between ^129^Xe atoms and host‐cages.

Due to its fully filled outermost electron layer, Xe exhibits minimal reactivity with active substances, resulting in excellent biosafety for in vivo applications. However, this inertness also poses challenges in interacting with target analytes, rendering direct use of HP ^129^Xe MRI in molecular imaging less feasible. To overcome this limitation, hydrophobic “cages” functionalized with targeting moieties are introduced as hosts to capture Xe atoms (**Figure** **5**). When housed within the host‐cages, Xe atoms are situated in a specific microenvironment, generating a new MR signal that can be differentiated from free Xe and convey targeting information. Consequently, the combination of HP ^129^Xe's high sensitivity with the precise selectivity of functionalized host‐cages significantly lowers the detection limit to sub‐nanomole levels for ^129^Xe‐based MR biosensors.

**Figure 6:**
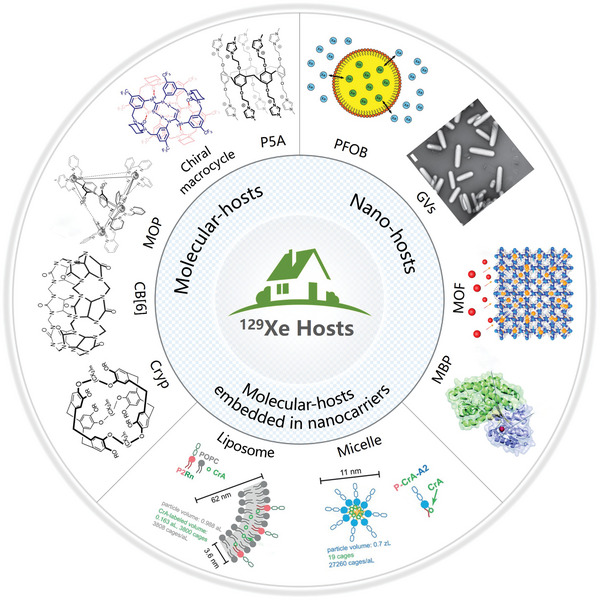
Different types of host cages for xenon atoms. There are three main categories, one for molecular‐hosts, another for nano‐hosts, and a combination of the two, which embeds the molecular hosts in a nanocarrier. Reproduced with permission.^[^ ^116^ ^]^ Copyright 2006, American Chemical Society. Reproduced with permission.^[^ ^117^ ^]^ Copyright 2015, Royal Society of Chemistry. Reproduced with permission.^[^ ^118^ ^]^ Copyright 2008, Wiley. Reproduced with permission.^[^ ^119^ ^]^ Copyright 2020, Springer Nature. Reproduced with permission.^[^ ^120^ ^]^ Copyright 2020, American Chemical Society. Reproduced with permission.^[^ ^121^ ^]^ Copyright 2013, American Chemical Society. Reproduced with permission.^[^ ^122^ ^]^ Copyright 2014, Springer Nature. Reproduced with permission.^[^ ^123^ ^]^ Copyright 2020, PNAS. Reproduced with permission.^[^ ^124^ ^]^ Copyright 2024, American Chemical Society. Reproduced with permission.^[^ ^125^ ^]^ Copyright 2020, Wiley‐VCH, Weinheim.

The affinity between Xe and the host‐cage is crucial for constructing an efficient molecular probe capable of generating its unique ^129^Xe MR signal. Variations in the structure of these hosts influence their affinity with ^129^Xe atoms, resulting in differing responses from the large polarizable electron cloud and manifesting as discrepancies in MR signals. So far, the utilized host‐cages can be roughly classified into two types: supramolecular hosts and nano‐hosts (**Figure** **6**). Supramolecular hosts, such as cryptophane and cucurbit[n]urils (**Table** **2**), possess a well‐defined intramolecular cavity structure with diameter slightly larger than that of a Xe atom (4.3 Å).^[^ ^126^ ^]^ These molecular hosts offer a specific cavity space capable of capturing a defined number of xenon atoms (Figure 5a). By comparison, nano‐hosts possess hydrophobic cores ranging in size from tens to hundreds of nanometers, providing heterogeneous spaces for hosting a large quantity of Xe atoms. Thus, the indefinite cavity spaces of nano‐hosts originating from the nonuniform particle sizes leads to an incalculable number of Xe atoms within the cage environment. Consequently, the exchange process of xenon atoms with nanocages is considerably more complex than that with molecular hosts, resulting in variations in hyperpolarized MR signals. Furthermore, some nanoparticles are served as carriers to load molecular cages, enhancing biocompatibility and targeting capabilities rather than directly generating new Xe MR signals.

**Table 2:** ^129^Xe atoms could be captured by molecules and nanomaterials with suitable cavities, generating specific magnetic resonance signals that reveal the unique chemical environment of the host.

	^129^Xe hosts		Cavity diameter	Cavity size	Sampling method	Saturation frequency	^129^Xe exchange rate
**Molecular‐hosts**	Cryptophanes	Cryptophane‐A^[^ ^128^ ^]^	5.4 Å	82 Å^3^	^129^Xe NMR	62.3 ppm	16 ± 3 s^−1^
		Cryptophane‐1.1.1^[^ ^129^ ^]^	–	81 Å^3^	^129^Xe NMR	31.1 ppm	2.4 s^−1^
		Cryptophane 222 (R = CH_2_COOH)^[^ ^116^ ^]^	–	95 Å^3^	^129^Xe NMR	64 ppm^a)^	3.2 s^−1^
		Cryptophane 223 (R = CH_2_COOH)^[^ ^116^ ^]^	–	102 Å^3^	^129^Xe NMR	52 ppm^a)^	11 s^−1^
		Cryptophane 233 (R = CH_2_COOH)^[^ ^116^ ^]^	–	117 Å^3^	^129^Xe NMR	42 ppm^a)^	37 s^−1^
		Cryptophane 333 (R = CH_2_COOH)^[^ ^116^ ^]^	–	121 Å^3^	^129^Xe NMR	35 ppm^a)^	90 s^−1^
		Cryptophane 111 functionalized with six [(η5‐C5Me5)RuII]^[^ ^130^ ^]^	7.4 Å	–	^129^Xe NMR	308 ppm^a)^	13.1 s^−1^
	C_60_^[^ ^131^ ^]^		7.1 Å	–	^129^Xe NMR	179.2 ppm^a)^	–
	Cucurbit[n]urils (CB[n])	CB[5]^[^ ^132^ ^]^	4.4 Å^[^ ^133^ ^]^	82 Å^3[^ ^133^ ^]^	^129^Xe NMR	230 ppm^a)^	–
		CB[6]^[^ ^117^ ^]^	5.8 Å^[^ ^133^ ^]^	164 Å^3[^ ^133^ ^]^	^129^Xe NMR Hyper‐CEST	122 ppm^a)^	1470 s^−1^
		CB[7]^[^ ^134^ ^]^	7.3 Å^[^ ^133^ ^]^	279 Å^3[^ ^133^ ^]^	Hyper‐CEST	100 ppm^a)^	–
	pillararenes^[^ ^120^ ^]^		5.8Å	–	Hyper‐CEST	−77 ppm^b)^	–
	γ‐cyclodextrins + pseudorotaxanes 1^[^ ^135^ ^]^ γ‐cyclodextrins + pseudorotaxanes 2^[^ ^135^ ^]^		8.3 Å	176.2 Å^3^ 170.1 Å^3^	Hyper‐CEST	128 ppm^a)^	–
	Chiral bisurea‐bisthiourea macrocycles^[^ ^119^ ^]^		9.6 Å, 10.5 Å	72 Å^3^, 36 Å^3^	^129^Xe NMR	169.6 ppm^a)^	–
	Bridged resorcinarene cages^[^ ^136^ ^]^	ABR‐6	–	52 Å^3^	Hyper‐CEST	340 ± 44 ppm	52000 ± 3000 s^−1^
		PBR‐3	–	163 Å^3^	Hyper‐CEST	114 ± 1 ppm, 210 ± 20 ppm	28000 ± 9000 s^−1^
	Metal–organic polyhedral cages^[^ ^137^ ^]^ (Fe‐MOP and its three diastereomers)		–	118.4 Å^3^, 105.7 Å^3^, 96.0 Å^3^	Hyper‐CEST	13, 24, and 30 ppm^b)^	2.7–4300 s^−1^
	Fe_4_L_6_ metallosupramolecular cage^[^ ^138^ ^]^		–	141 Å^3^	^129^Xe NMR	204.3 ppm^a)^	10 s^−1^
	[Co4L6]4−[139]		–	135.3 Å^3^	Hyper‐CEST	−89 ppm^b)^	4.45 × 10^2^ s^−1^
	Co_n_Fe_4‐n_ Metal‐Organic Capsules^[^ ^140^ ^]^	[(Xe)CoFe_3_L_6_]^4−^ [(Xe)Co_2_Fe_2_L_6_]^4−^ [(Xe)Co_3_FeL_6_]^4−^ [(Xe)Co_4_L_6_]^4−^	–	–	^129^Xe NMR/ Hyper‐CEST	−22.6 ppm^b)^ −49.7 ppm^b)^ −72.8 ppm^b)^ −94.4 ppm^b)^	–
	Mesomeric bisurea‐bisthiourea macrocycle^[^ ^141^ ^]^		–	76 Å^3^ (M_S_)_2_/(M_R_)_2_ 77 Å^3^ (M_S_ M_R_)	^129^Xe NMR	148.4 ppm (M_S_M_R_) 165.1 ppm (M_S_)_2_ 166.6 ppm (M_R_)_2_	–
	Maltose‐binding protein (MBP)^[^ ^142^ ^]^		–	75–95 Å^3^	Hyper‐CEST	95 ppm^b)^	(8.6 ± 2.1) × 10^2^ s^−1^
	Ribose‐binding protein (RBP)^[^ ^143^ ^]^		–	–	Hyper‐CEST	233 ppm^a)^	–
	TEM‐1 β‐lactamase (Bla)^[^ ^144^ ^]^		–	–	Hyper‐CEST	255 ppm^a)^	–
**Molecular‐hosts** **loaded in nanoparticles**	Cryptophane‐A functionalized liposomes^[^ ^145^ ^]^		105–125 nm	–	Hyper‐CEST	−121 ppm^b)^	155 s^−1^
	Cryptophane‐A functionalized micelles^[^ ^125^ ^]^		≈11 nm	–	Hyper‐CEST	70 ppm^a)^	–
**Nano‐hosts**	Perfluorooctyl bromide (PFOB) emulsions^[^ ^146^ ^]^		1.5 ± 2.0 µm	–	^129^Xe NMR	106 ppm^a)^	11765 s^−1^
	PFOB nanoemulsions^[^ ^121^ ^]^		160–310 nm	–	Hyper‐CEST	111 ± 9 ppm^b)^	37 037 s^−1^ (160 nm) 20833 s^−1^ (210 nm) 13157 s^−1^ (265 nm) 9615 s^−1^ (310 nm)
	Metal–organic frameworks	ZIF‐8^[^ ^123^ ^]^	3.8–4.3 Å	–	^129^Xe NMR	82–88 ppm^a)^	–
		IRMOF‐1^[^ ^147^ ^]^	7.93 Å	–	Hyper‐CEST	48 ppm^b)^	429 ± 152 s^−1^
		IRMOF‐8^[^ ^147^ ^]^	9.17 Å	–	Hyper‐CEST	17 ppm^b)^	1176 ± 937 s^−1^
		IRMOF‐10^[^ ^147^ ^]^	12.15 Å	–	Hyper‐CEST	26 ppm^b)^	2496 ± 548 s^−1^
		MOL^[^ ^124^ ^]^	1.19 nm	–	Hyper‐CEST	204 ppm^a)^	2450 ± 694 s^−1^
	Gas vesicles^[^ ^148^ ^]^	Ana	519 nm	–	Hyper‐CEST	−176.2 ± 0.1 ppm^b)^	19300 ± 1800 s^−1^
		Halo	400 nm	–		−181.7 ± 0.5 ppm^b)^	15300 ± 1500 s^−1^
		Mega	250 nm	–		−155.7 ± 2.1 ppm^b)^	22600 ± 2800 s^−1^

^a)^ Gaseous ^129^Xe is the reference at 0 ppm;

^b)^ Dissolved ^129^Xe is the reference at 0 ppm.

**Figure 7:**
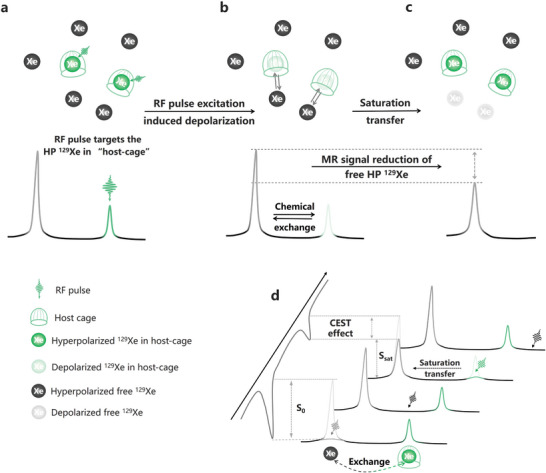
Hyperpolarized ^129^Xe chemical exchange saturation transfer (Hyper‐CSET): Amplifying the target ^129^Xe within “host‐cages” by indirectly detecting the reduction in signal of free HP ^129^Xe. a) An RF pulse is applied to target the HP ^129^Xe in host‐cages, resulting in depolarization of these ^129^Xe atoms and saturation of the corresponding MR signal. b) Subsequently, the ^129^Xe atoms within the host‐cages exchange with the free HP ^129^Xe atoms, c) leading to a reduction in MR signal of the free HP ^129^Xe due to the accompanying saturation transfe. d) A Hyper‐CSET spectrum is obtained by comparing the reduction in MR signal from free HP ^129^Xe atoms before and after the application of a saturation pulse, which reflects the specific MR signal generated by HP ^129^Xe within host‐cages.

The dynamic behavior of ^129^Xe atoms within host cages facilitates their integration with another amplification strategy known as chemical exchange saturation transfer (CEST), which significantly enhances the MR signal by several orders of magnitude. Essentially, this strategy capitalizes on the distinguishable MR signals between HP ^129^Xe atoms confined within host‐cages and those freely present in the surrounding environment. Specifically, radiofrequency (RF) pulses are precisely applied to target the resonance frequency of the HP ^129^Xe atoms within hosts (**Figure** **7**a), causing their depolarization (Figure 7b). Subsequently, these depolarized ^129^Xe atoms exchange with the free HP ^129^Xe atoms in the environment. As a result, the number of free HP ^129^Xe atoms in the environment decreases (Figure 7c) after exchange, which reduces the corresponding HP MR signal. By comparing the MR signals of HP ^129^Xe atoms before and after RF pulse saturation, the encapsulated ^129^Xe within host cages can be characterized by the diminished MR signal from free HP ^129^Xe atoms (Figure 7d). The extent of loss in the MR signal generated by free HP ^129^Xe atoms is directly proportional to the concentration of the cages. Through the synergistic combination of the exceptional sensitivity of HP ^129^Xe and the signal amplification of CEST, a novel approach known as hyperpolarized ^129^Xe chemical exchange saturation transfer (Hyper‐CSET) is established, elevating the detection limit to picomole levels,^[^ ^127^ ^]^ which is six orders of magnitude higher than direct detection of the bound ^129^Xe signal.

### 5.1 Molecular Hosts‐Based ^129^Xe Biosensors

Due to its large, polarizable electron cloud, ^129^Xe is highly sensitive to transient binding events, leading to changes in MR signals that reflect the chemical environment of the cavity in which ^129^Xe atoms are located. Molecular cavities, such as cryptophanes, cucurbit[n]urils, pillararenes, metal–organic polyhedra, and mesomeric bisurea‐bisthiourea macrocycles, provide distinct chemical environments for ^129^Xe atoms, resulting in unique MRI signals ranging from 30 to 340 ppm (Table 2). These specific MR signals can be utilized for molecular sensing, especially when the molecular hosts are further modified with targeting segments (Figure 5a).

#### 5.1.1 Cryptophanes

**Figure 8:**
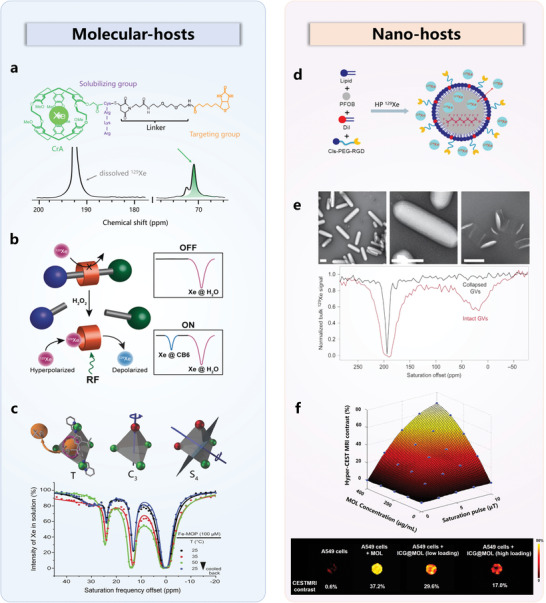
Functionalization and applications of ^129^Xe hosts. a) CrypA was functionalized with a solubilizing group (purple) and a targeting group (yellow) via a rink amide resin linker. Adapted with permission.^[^ ^149^ ^]^ Copyright 2001, PNAS. b) A “turn‐on” strategy for target analysis using a CB[6]‐based probe. Initially, the cavity of the CB[6] host was occupied by rotaxane molecules. Upon encountering the target molecule H_2_O_2_, the rotaxane was cleaved, freeing the host cavity for HP ^129^Xe exchange. As a result, the specific ^129^Xe MR signal for CB[6] was recovered with the introduction of the target molecules. Reproduced with permission.^[^ ^158^ ^]^ Copyright 2019, Wiley. c) The ultrasensitive property of HP ^129^Xe allows for the differentiation of diastereoisomers of Fe‐MOP. Reproduced with permission.^[^ ^137^ ^]^ Copyright 2022, Springer Nature. d) PFOB nano‐emulsions were functionalized with RGD peptides to target tumor cells, and resulting systems were imaged using ultrasensitive ^129^Xe MRI. Reproduced with permission.^[^ ^159^ ^]^ Copyright 2019, American Chemical Society. e) GVs displayed a characteristic signal peak only when their structure remains intact; this peak vanished when the structure collapses. Reproduced with permission.^[^ ^122^ ^]^ Copyright 2014, Springer Nature. f) Establishment of a 3D map to illustrate the relationships among the ^129^Xe CEST MRI contrast, MOL concentration, and saturation pulse, with its application in quantifying MOL‐hosts and the content of drug molecules within living cells. Reproduced with permission.^[^ ^124^ ^]^ Copyright 2024, American Chemical Society.

The original cryptophane, first synthesized in 1981, comprised of two cyclotriveratrylene (CTV) caps connected by three ethyl linkers. However, it was not until 1998 that cryptophane‐A was reported to reversibly trap xenon within its cavity, with a stability constant *K*_A_ ≈ 3000 m ^−1^ in C_2_D_2_Cl_4_ at 278 K.^[^ ^128^ ^]^ Cryptophane‐A forms a 1:1 host–guest complex with xenon, resulting in a strong MR signal that could be directly detected and distinctly separated from the free and captured xenon in ^129^Xe NMR spectrum. The first cryptophane‐based ^129^Xe biosensor was developed by Pines and co‐workers in 2001,^[^ ^149^ ^]^ in which a peptide modified bridge was used to link cryptophane‐A with a biotin moiety with significantly improved the water solubility (**Figure** **8**a). When 80 nmol of avidin was added, the MR signal corresponding to ^129^Xe in cryptophane‐A shifted 2.3 ppm downfield, indicating the binding of biotin to avidin. Further study revealed that interaction with the protein led to deformation of the cryptophane host and distortion of xenon's electron cloud.

This groundbreaking research not only confirms the high sensitivity of HP ^129^Xe NMR, which improves detection limit from sub‐millimolar to nanomolar levels, but also provides a universal strategy for detecting a wide range of biomarkers. Theoretically, biotin can be replaced with other target moiety capable of binding or interacting with analyte of interest. The target moiety is often modified with an amino group and then linked to a carboxylated cryptophane via amide condensation, or it can be an azido group that reacts with alkynyl cryptophane via [3+2] azide‐alkyne cycloaddition. In addition, one or more solubilizing groups, such as carboxylic acids, can be introduced to improve the water solubility and biocompatibility of hydrophobic cryptophanes. Using this strategy, Dmochowski and colleagues developed a cryptophane‐based xenon biosensor targeting integrin receptors.^[^ ^150^ ^]^ They conjugated a cyclic RGDyK peptide affinity tag to cryptophane‐A via a propargyl group using cycloaddition reaction and introduced two propionic acid group to the conjugate to enhance water solubility. The ^129^Xe MR signal shifted 4.1 ppm downfield when the biosensor specifically recognized the *α_v_β_3_* integrin. They also used Alexa Fluor 488 dye to label the biosensor for auxiliary validation. Later, they made significantly advancements and successfully developed multiple biosensors for detecting various targets, including carbonic anhydrase,^[^ ^151^ ^]^ matrix metalloproteinase‐7,^[^ ^152^ ^]^ and Calmodulin.^[^ ^153^ ^]^

Kotera et al.^[^ ^154^ ^]^ designed a cryptophane‐based sensor by incorporating a zinc‐chelating group to cryptophane, enabling the measurement of Zn^2+^ at concentrations as low as 100 × 10^−9^ m. Berthault et al.^[^ ^155^ ^]^ functionalized cryptophane with a 20‐mer nucleotide, observing a 1.5 ppm upfield shift signal in Hyper‐CEST when the oligonucleotide strand bound to its complementary DNA strand, whereas no change occurred in the presence of a noncomplementary strand. They also achieved accurate pH measurement using a pair of pH‐sensitive cryptophane‐based sensors.^[^ ^156^ ^]^ By measuring the difference between their chemical shifts, measurement error caused by temperature, ionic strength, and other factors were eliminated. Furthermore, an acrylate group was linked to cryptophane to serve as a reactive site for covalently bonding biothiols,^[^ ^157^ ^]^ aiming to monitor thiol‐addition reaction via Hyper‐CEST. This biosensor allowed for the discrimination of cysteine, homocysteine and glutathione based on chemical shift and average reaction rate.

#### 5.1.2 Cucurbit[n]uril

Although cryptophanes have shown significant potential in capturing ^129^Xe atoms and generating specific magnetic resonance signals for highly sensitive biosensing, the complex synthesis, low productivity, and poor water solubility continue to limit their broader application in biological systems. Therefore, it is crucial to explore alternative cages that can serve as ^129^Xe MR signal reporters with easier accessibility, better biocompatibility, and commercial availability.

Cucurbit[6]uril (CB[6]) is a hexameric macrocyclic compound that can be easily synthesized from the condensation of glycoluril and formaldehyde catalyzed by acid.^[^ ^160^ ^]^ The structure of CB[6] resembles a pumpkin and features a hydrophobic cavity with a diameter of ≈5.5 Å, similar in size to cryptophane. Guest molecules can enter the cavity through two opposing quasi‐planar portals decorated with carbonyl groups.^[^ ^161^ ^]^ It has been reported that that the affinity between CB[6] and xenon is moderate, meeting the requirements for detection by Hyper‐CEST.^[^ ^117^ ^]^ Direct sampling ^129^Xe NMR revealed a signal shifted 72 ppm upfield from the free xenon (192 ppm), indicating the presence of ^129^Xe within CB[6] cavity when 5 × 10^−3^ m CB[6] was dissolved in a buffer at pH 7.2. The association constant K_A_ for xenon and CB[6] in PBS at pH 7.2 is 490 m ^−1^ at 300 K, which is considerably lower than that of cryptophane.^[^ ^130^, ^162^ ^]^ However, the exchange rate of ^129^Xe in CB[6] is ≈17 times higher than in triacetic acid cryptophane (κ_exch_ = 86 s^−1^) at 300 K.^[^ ^127^ ^]^ This rapid exchange resulted in broadening of the line shape for both signals from free ^129^Xe and ^129^Xe in CB[6]. Nevertheless, an ultralow detection limit of 1.8 × 10^−12^ m CB[6] was achieved by applying shaped RF saturation pulses at the chemical shift of ^129^Xe in CB[6] and measuring the residual aqueous ^129^Xe signal after spin transfer as on‐resonance CEST response.

CB[6]‐based sensors are primarily designed using a “turn‐on” strategy (Figure 8b). In this approach, the cavity of CB[6] is first occupied by a guest molecule, which prevents the access of ^129^Xe atoms and locks the accompanying MR signal. When induced by the target analyte, the guest molecule is displaced, freeing space within the cavity. This activation facilitates a host–guest interaction between CB[6] and the ^129^Xe atoms, resulting in a recovered signal. Consequently, the MR signal from ^129^Xe within CB[6] remains suppressed until activated by target molecules, providing a more pronounced off‐on signal in complex biological environments compared to measuring small chemical shift differences between the hosted and free states.

For instance, Dmochowski et al.^[^ ^163^ ^]^ developed a CB[6]‐based molecular relay for detecting specific proteins within a complex mixture. This molecular relay was programmed for three sequential recognition events: initially, a two‐faced guest (TFG) initially bound CB[6], blocking the Xe‐CB[6] exchange; then, the TFG was sequestered by protein analyte, causing the release of CB[6]; finally, ^129^Xe atoms entered the CB[6] cavity, generating a specific signal for ^129^Xe@CB[6] that was monitored by Hyper‐CEST. The TFG played a crucial role in the relay by bonding CB[6] with intermediate affinity, ensuring xenon displacement and could be readily sequestered after the opposite face bound to a higher‐affinity target protein.

Francis et al.^[^ ^164^ ^]^ designed a chemically activated sensor in which CB[6] was mechanically locked in a rotaxane structure, with both ends of the axle capped by bulky stopper groups that prevented ^129^Xe from accessing the CB[6] cavity. One of the bulky stoppers was engineered to cleave under specific conditions, thereby freeing the CB[6] cavity for ^129^Xe accessing and generating a distinct ^129^Xe@CB[6] signal. Initially, no such signal was observed even at relatively elevated concentrations of the CB[6]‐rotaxane complex. However, upon treatment with 10 equivalent of LiOH to catalyze ester hydrolysis, CB[6] was released, leading to a notable ^129^Xe@CB[6] signal. Leveraging this approach, they successfully identified the disease‐related enzyme matrix metalloprotease 2.^[^ ^165^ ^]^

#### 5.1.3 Metal–Organic Polyhedras

Metal–organic polyhedra (MOP) is discrete supramolecular metal complex formed through the self‐assembly of metal cations and organic ligands. These metal complexes can create hydrophobic cavities of diverse structures, facilitating the encapsulation of hydrophobic guests in both aqueous and organic environments. By altering metal nodes and organic ligands, the MOP cavity can be finely tuned to accommodate the size of xenon atoms.

Fe_4_L_6_ is a tetrahedral structure formed through the self‐assembly of 4,4′‐diaminobiphenyl‐2,2′‐disulfonic acid, 2‐formylpyridine, and Fe(II) ions (Figure 8c).^[^ ^118^, ^137^ ^]^ With its 12 sulfonate groups, Fe_4_L_6_ exhibits excellent water solubility (34 g L^−1^), enabling it to encapsulate ^129^Xe atoms in water. Roukala et al.^[^ ^138^ ^]^ observed that ^129^Xe inside the Fe_4_L_6_ shifted 16.6 ppm downfield compared to free ^129^Xe in water. The exchange rate between encapsulated and free ^129^Xe was ≈10 Hz, making it suitable for CEST experiments. In addition, the shift of ^129^Xe within Fe_4_L_6_ was found to be linearly dependent on temperature, increasing at a rate of 0.042 ppm K^−1^.

Co_4_L_6_ is another MOP used as a host for ^129^Xe. Recent study revealed that the exchange rate of ^129^Xe atoms in Co_4_L_6_ was significantly faster than that in its Fe_4_L_6_ counterpart,^[^ ^139^ ^]^ exhibiting a 40‐fold higher dissociation constant and generating a substantial Hyper‐CEST signal at 89 ppm, compared to solvated free ^129^Xe. Furthermore, the electron spin hyperfine interactions between the Co metal centers at the vertices of Co_4_L_6_ tetrahedron and the trapped ¹^2^⁹Xe atoms resulted in a temperature‐dependent shift with a slope of −0.41 ppm K^−1^. A series of bimetallic intercalation‐doped Co*_n_* Fe_4−_ *_n_* L_6_ metal–organic cages were also explored,^[^ ^140^ ^]^ demonstrating excellent ¹^2^⁹Xe sensing capabilities. These findings highlight the tunability of M_4_L_6_ systems and their significant potential for developing ¹^2^⁹Xe MR sensors.

#### 5.1.4 Proteins

Linear peptide chains fold to form functional proteins with specific three‐dimensional structures, a process that is complex and highly coordinated. During folding, peptide chains create hydrophobic cavities, a key feature of protein formation. By sequestering hydrophobic residues inside the protein, the free energy of the system is reduced, which is crucial for the stability of the resulting protein structure. These cavities play a vital role in molecular binding, catalytic activity, and functional regulation. Notably, the high affinity of xenon atoms for hydrophobic cavities makes these sites potential binding locations for xenon during protein folding. This interaction may offer valuable insights into protein structure and function.

It has been demonstrated that TEM‐1 β‐lactamase from E. coli could be used as a single‐protein contrast agent for Hyper‐CEST in both bacterial and mammalian cells.^[^ ^144^ ^]^ The transient binding of xenon to the primary allosteric site of TEM‐1 β‐lactamase produced a distinct signal peak at 255 ppm, suggesting that HP ^129^Xe could serve as an ultrasensitive probe for studying allosteric pockets in proteins. Following this, maltose‐binding protein (MBP) was developed as a genetically encoded HP ^129^Xe MRI probe, allowing quantification of maltose at nanomolar concentrations.^[^ ^142^ ^]^ When maltose bond to MBP, the protein's conformation changed, resulting in a slower exchange rate of ^129^Xe and a signal at 95 ppm downfield compared to solvated free ^129^Xe.

Protein probes are highly specific, capable of precisely recognizing and binding to target molecules, cellular receptors, or other proteins, making them effective tools for studying specific processes in complex biological systems. More importantly, the hydrophobic pocket of proteins can be precisely designed and modified through genetic engineering to meet various functional needs. As naturally occurring substances in living systems, protein probes are inherently compatible with living organisms, which helps minimizing non‐specific reactions and side effects. HP ^129^Xe NMR and MRI provide novel and powerful means for studying the binding process between a protein's active site and its specific substrate. These techniques are particularly suited for sensitive, lossless, in situ, and real‐time analysis with minimal background noise.

### 5.2 Molecular Hosts Loaded by Nanoparticles

To date, most of the ^129^Xe‐based sensors have been developed using supramolecular host‐cages, primarily including cryptophanes and cucurbit[n]uril. However, molecular modification often requires multiple chemical reactions, particularly when integrating several functional groups into one molecule, resulting in a complex synthesis process. Fortunately, the introduction of nanomaterials in drug delivery,^[^ ^166^ ^]^ coupled with their ease of functionalization, provides a promising alternative to overcome the limitations of molecular hosts. Lipid nanoparticles are initially considered for their high encapsulation efficiency to load both hydrophilic and hydrophobic molecules.

A straightforward preparation strategy involves embedding molecular hosts within the phospholipid bilayer of liposomes and attaching recognition modules to the nanoparticles’ surface. For instance, a single POPC‐based liposome could accommodate ≈3800 Cryptophane‐A (CrA) units while preserving its structural integrity.^[^ ^167^ ^]^ The introduction of recognizing modules only slightly increased liposome's particle size, and the toxicity was significantly reduced compared to naked CrA administration. Fluorescence co‐localization studies subsequently confirmed that the liposome structure remained intact without degradation for up to 4 hours. This demonstrates the feasibility of using liposomes as delivery vehicles for ^129^Xe biosensors, due to their efficient loading capacity, specific targeting ability, and good biocompatibility—making them ideal for applying in biological systems. Another typical example is to integrate amphiphilic cryptophane onto the surface of nanoemulsions along with targeting and fluorescence modules.^[^ ^168^ ^]^ This successful integration enabled tumor‐targeted multimodal imaging (FL, ^19^F MRI, ^129^Xe Hyper‐CEST MRI) combined with photodynamic therapy.

Beyond lipid nanoparticles, micelles have also been used to load cryptophane for targeted ^129^Xe MRI.^[^ ^125^ ^]^ Although nanoparticle loading provides a simple solution to fabricate supermolecule‐based ^129^Xe biosensors, their application is currently limited at cellular levels. Still, there is a need to explore more suitable nanocarriers that could advance HP ^129^Xe MRI for in vivo molecular imaging applications.

### 5.3 Nanoparticles as Hosts to Capture ^129^Xe Atoms and Generate Corresponding MR Signals

Some nanoparticles possess specific cavity structures that can isolate their unique chemical environments from the surrounding dispersion medium. These nanoparticles can provide a host space to capture ^129^Xe atoms and generate corresponding MR signals to indicate their host state. To date, there are two types of nano‐hosts for ^129^Xe:

1. Nano‐hosts with cavity diameters in the range of hundreds of nanometers, which offer a large space for ^129^Xe. In these hosts, the exchange rate is relatively rapid, and the resulting specific signal is usually determined by Hyper‐CEST.

2. Nano‐hosts with well‐defined cavity structures on the order of several nanometers, where the exchange of ^129^Xe can be precisely controlled. This allows for MR signals to be detected either by directly sampling ^129^Xe NMR or indirectly sampling Hyper‐CEST.

#### 5.3.1 PFOB Nanoemulsions

Perfluorooctyl bromide (PFOB), a member of the perfluorocarbons (PFCs) family, is widely used in ^19^F MRI^[^ ^169^ ^]^ and ultrasound imaging^[^ ^170^ ^]^ for tumor imaging and treatment in vivo. One of PFOB's most significant advantages is its excellent gas solubility. Notably, PFOB nanoemulsion, which is dissolved with oxygen, can serve as a blood substitute.^[^ ^171^ ^]^ In addition, the solubility of ^129^Xe in PFOB is ten times higher than in water. Studies have shown that PFOB nanoemulsion can be used as a carrier for intravenous delivery of hyperpolarized ^129^Xe.^[^ ^146^ ^]^ Importantly, ^129^Xe dissolved in PFOB exhibits a specific chemical shift of ≈100 ppm, distinct from the ≈192 ppm shift of ^129^Xe dissolved in aqueous solutions. Compared with cryptophane, PFOB nanoemulsion contains a much higher concentration of xenon atoms per droplet and the allows for faster xenon exchange between droplets and the surrounding medium, thereby enhancing contrast per agent.

In 2013, Pines et al. used a poloxamer surfactant (Pluronic F‐68) to produce PFOB nanoemulsion with a narrow size distribution (160–310 nm).^[^ ^121^ ^]^ Hyper‐CEST spectrum revealed two signals: a broad peak at (111 ± 9) ppm corresponding to ^129^Xe dissolved in PFOB and another peak at 192 ppm corresponding to free ^129^Xe in aqueous solutions. It was demonstrated that as the sizes of the nanoemulsion increased, the xenon exchange rate slowed due to diffusion‐limited residence times, with larger nanoemulsion exhibiting a stronger CEST effect than small ones. Shortly thereafter, Schröder et al.^[^ ^172^ ^]^ successfully labeled cells with PFOB nanoemulsion and achieved intracellular detection using Hyper‐CEST at nanomolar concentrations.

Our team developed a PFOB nanoemulsion‐based probe that integrated ^129^Xe Hyper‐CEST MRI, ^19^F MRI, and fluorescent imaging for selectively detecting tumor cells (Figure 8d).^[^ ^159^ ^]^ Using phospholipids as emulsifier, PFOB nanoemulsion with an average diameter of 195 nm was prepared, which provided strong ^19^F MRI signal due to the high concentration of ^19^F in PFOB. In addition, ^129^Xe atoms entered into the unique chemical environment of PFOB nanoemulsion, generating a specific signal for ^129^Xe Hyper‐CEST MRI. Furthermore, a fluorescent dye (DiI) was incorporated into the phospholipid surface of PFOB nanoemulsion via hydrophobic interaction, equipping the probe with fluorescent imaging function. A cholesterol‐labeled peptide (Cls‐PEG‐RGDyc) was also introduced to enhance targeting capabilities of the nanoemulsion. This multimodal imaging system has been demonstrated to detect tumors cells selectively and sensitively across molecular, cellular, and animal levels.

However, the relatively fast exchange of ^129^Xe in the PFOB nanoemulsion necessitates high‐power saturation pulses for efficient saturation, which poses a risk of overheating in biological applications. In addition, variability in particle sizes of the PFOB nanoemulsion over time may limit its further application. To address these issues, we moderated the exchange rate of ^129^Xe in PFOB nanoemulsions by adjusting pore sizes of the outer layer porous silica shells.^[^ ^173^ ^]^ The pores in the silica shell, with an average diameter of 6.9 nm, acted as channels for ^129^Xe to enter and exit the PFOB liquid core. In Hyper‐CEST results, the signal corresponding to ^129^Xe dissolved in PFOB was observed at around 106 ppm, with a narrow line width compared to previously reported PFOB nanoemulsions,^[^ ^121^, ^146^ ^]^ providing a well‐resolved signal and low detection threshold.

#### 5.3.2 Genetically Encoded Gas Vesicles

Gas vesicles (GVs) are subcellular structures found almost exclusively in aquatic microorganisms.^[^ ^174^ ^]^ They consist of a hollow interior filled with gas and an outer protein shell.^[^ ^175^ ^]^ The vesicle shells are primarily composed of a hydrophobic protein, GvpA, which is highly permeable to gas molecules. Intracellular GVs act as flotation devices by providing buoyancy, allowing water‐borne microorganisms to float toward the surface in search of light and nutrients.^[^ ^176^ ^]^ Additionally, photosynthetic bacteria can produce carbohydrates that serve as ballasts through photosynthesis. The combined effects of GVs for buoyancy and carbohydrate ballast enable diurnal vertical migrations.^[^ ^177^ ^]^ Typically, fully formed GVs are spindle‐ or cylinder‐shaped, ranging from 0.1 to 2 µm in length, with width varies from 45 to 250 nm.^[^ ^178^ ^]^ Currently, the most extensively studied GVs are those from cyanobacterium, *Anabaena flos‐aquae* and the haloarchaea.

Initially, GVs were used as ultrasound contrast agents. They scatter sound waves efficiently, creating strong ultrasound contrast due to the acoustic impedance mismatch between the gas interior of GVs and the surrounding aqueous media.^[^ ^179^ ^]^ Shapiro et al. demonstrated that GVs could serve as a promising class of Hyper‐CEST contrast agents (Figure 8e).^[^ ^122^ ^]^ Results indicated that HP ^129^Xe atoms dissolved in the aqueous phase would diffuse into GVs, forming a gaseous phase with a distinct chemical shift (≈31.2 ppm) and rapidly exchanging between the GVs and the solution. Furthermore, GVs from different species exhibited different chemical shifts due to their varying shapes and sizes. For instance, the CEST signals for GVs derived from *Halobacteria sp*. NRC‐1, *Microcystis sp*., and *E. coli* were observed at 14.4, 30.6, and 51.4 ppm, respectively. This variability enabled multiplexing through saturation at different frequencies. In addition, targeting moiety was successfully attached to the surface of GVs, allowing for the specific labeling of breast cancer cells.

Admittedly, these gas‐filled, protein‐shelled nanostructures exhibited certain advantages over PFOB nanodroplets in terms of xenon cargo capacity, which could be detected even at picomolar concentrations using Hyper‐CEST.^[^ ^180^ ^]^ Different from other Xe hosts, the excellent CEST performance of GVs only relied on the physical partitioning of the dissolved ^129^Xe gas. The loading capacity of GVs is elastic: the xenon fraction partitioning into gas vesicles followed the ideal gas law, which provided stable CEST contrast at varying concentrations of xenon.^[^ ^148^ ^]^ Consequently, the efficiency of the CEST pool maintained even as the bulk pool increased, thereby enhancing the signal‐to‐noise ratio.

#### 5.3.3 Tailored Metal–Organic Frameworks

Metal–organic frameworks (MOFs) are crystalline porous solids,^[^ ^181^ ^]^ composed of metal ions or clusters and organic ligands, which have gained significantly interest in fields such as energy storage and conversion,^[^ ^182^ ^]^ catalysis,^[^ ^183^ ^]^ molecular recognition,^[^ ^184^ ^]^ and drug delivery.^[^ ^185^ ^]^ Compared with other inorganic porous nanomaterials, MOFs offer attractive advantages due to their customized skeleton, structure, and pore size, which can be carefully tuned by the starting derivatives and metals. Early research frequently utilized ^129^Xe NMR to characterize porous materials like MOFs, providing valuable information on their surface and pore system through measurements of chemical shifts, line widths, longitudinal relaxation times (T_1_) and other parameters.^[^ ^186^ ^]^ Importantly, the interaction between xenon and MOF has facilitated the development of ^129^Xe biosensors.

Our team introduced ZIF‐8 nanoparticles composed of Zn (II) as the metal nodes and 2‐methylimidazole as the organic linker, which served as nano‐hosts for capturing ^129^Xe.^[^ ^123^ ^]^ When dispersed in aqueous solution, the free dissolved ^129^Xe interacted specifically with the hydrophobic cavity of ZIF‐8, generating a characteristic signal at a chemical shift of 84 ppm. We demonstrated that the ^129^Xe signal intensity in ZIF‐8 (110 nm in size, at a concentration of 100 mg mL^−1^) was four times greater than that of the dissolved free ^129^Xe and 200 times greater than that of CrA under identical experimental conditions. This indicated the strong capability of the hydrophobic cavity in ZIF‐8 to capture ^129^Xe. As a result, ZIF‐8 nanoparticles were detectable via direct sampling ^129^Xe NMR with a high signal‐to‐noise ratio when dispersed in aqueous solutions.

Subsequently, we aimed to manipulate the pore structure of MOF to create editable chemical environments for ^129^Xe guesting, leading to tunable MR signals. By utilizing zinc ions as the metal center, we manipulated the length of the organic ligands, which shaped the pore diameters and structures.^[^ ^147^ ^]^ The resulting MOFs provided a variety of chemical microenvironments, each of which hosted ^129^Xe and generated specific MR signals that reflect the local environments. Specifically, signal peaks at 48, 17, and 26 ppm corresponded to IRMOF‐1, IRMOF‐8, and IRMOF‐10, respectively. Inspired by the way quantum dots of different particle sizes fluoresce in various colors, we visualized the ^129^Xe signals in different MOFs by assigning specific colors to each chemical shift. This study addresses the long‐standing multiplexing problem in ^129^Xe MRI.

### 5.4 Molecular Detection in Cellular Levels Using ^129^Xe‐Based Biosensors

Once a host‐cage capable of capturing ^129^Xe generates a specific MR signal, it can readily participate in acting as a signal unit to fabricate a biosensor for cellular detection. The complexity of the cellular environment, differences in specific molecules, and diversity of biological reactions can serve as switches for biosensor accumulation or signal activation. Furthermore, the proposed Hyper‐CEST method advances the application of ^129^Xe‐based biosensors from solution to cellular levels.

For example, Schröder et al. employed Hyper‐CEST to monitor the intracellular cryptophane.^[^ ^187^ ^]^ The chemical shift difference between free ^129^Xe and ^129^Xe within the cell‐internalized cryptophane was substantial, facilitating saturation‐transfer‐based MRI experiments. Establishing on this phenomenon, they successfully implemented HP ^129^Xe MRI of cell‐surface receptors^[^ ^188^ ^]^ and cell‐surface glycans^[^ ^189^ ^]^ by linking cryptophane with corresponding targeting moieties. Recently, our group developed a series of RGD peptide modified nanoemulsions that specifically recognized integrins overexpressed on the cell surface, leading to the generation of specific HP ^129^Xe MRI results for monitoring target non‐small‐cell lung cancer A549 cells.^[^ ^159^, ^168^, ^173^ ^]^

In addition to designing binding sites for identifying cell surface receptors, intracellular organelles and active substances are also considered as potential target sites. Our team developed a mitochondria‐targeting biosensor that enabled the detection of intracellular biothiols using Hyper‐CEST.^[^ ^190^ ^]^ This biosensor featured cryptophane‐A as the ^129^Xe MR signal reporter, linked to a naphthalimide fluorescence reporter and a triphenylphosphonium targeting unit via a disulfide bond. Exposure to biothiols cleaved the disulfide bond, which activated the fluorescence and caused a 1.4 ppm upfield shift in Hyper‐CEST. By combining the high sensitivity of HP ^129^Xe and the dual targeting of intracellular mitochondrion and biothiol, we improved the detection limit to 200 pM with Hyper‐CEST, leading the sensitivity of magnetic resonance‐based method to a new level. A CB[6]‐based biosensor was designed using a “turn‐on” strategy to monitor the expression of intracellular enzyme. The strong noncovalent interactions between amino‐groups and CB[6] could lock the cavity with putrescine dihydrochloride, while the specific ^129^Xe MR signal in small intestinal villus epithelial cells was activated when intracellular overexpressed diamine oxidase catalyzed the breakdown of putrescine dihydrochloride, releasing the CB[6]‐cavity.^[^ ^191^ ^]^ Furthermore, certain intracellular proteins could be directly detected due to capability to host ^129^Xe within their hydrophobic pockets. For instance, ribose‐binding proteins (RBP) were used as ^129^Xe biosensors to measure ribose levels in mammalian cell lysates and serum. The binding of ribose induced a “closed” conformation in the RBP, which slowed ^129^Xe exchange to a rate detectable by Hyper‐CEST.^[^ ^143^ ^]^

In addition, HP ^129^Xe MRI can be utilized to monitor the metabolism of endogenous substances in cells by rationally designing probes, such as those for intracellular H_2_O_2_,^[^ ^158^ ^]^ H_2_S^[^ ^192^ ^]^ levels, and pH.^[^ ^193^ ^]^ During the onset and progression of disease, endogenous substances within cells are often imbalanced, which may impair cellular function and provide conducive conditions for disease development. Monitoring changes in these endogenous substances could facilitate a deeper understanding of disease mechanisms and the identification of potential biomarkers. This, in turn, would provide valuable insights for disease prevention, early diagnosis and treatment.

Moreover, exogenous drugs uptake by cells were readily quantified using HP ^129^Xe MRI. Recently, we developed a novel approach to alter the chemical microenvironment of pore cavities in 2D lamellar MOF by embedding drug molecules.^[^ ^124^ ^]^ The introduction of guest drug molecules reduced the free space within the pore cavities, thereby hindering the access of ^129^Xe atoms and weakening the ^129^Xe MR signals. Notably, changes in the contrast of Hyper‐CEST signals exhibited a linear relationship with the loading content of drug molecules in the MOF. Based on this relationship, we draw a 3D map (Figure 8f) to establish a new method for quantitatively assessing the amount of MOF carriers and the uptake drug molecules in living cells using Hyper‐CEST MRI. Remarkably, the resulting ^129^Xe MRI signals showed similar responses to drug loading for a given loading value, regardless of whether the drug was delivered with the nanocarrier or if the drug was exposed to the cells afterwards. This highlights the potential application of the ^129^Xe‐based microenvironmentally responsive method in complex biological systems.

### 5.5 Cages Applied for In Vivo HP ^129^Xe MRI

Detection of probe under in vivo conditions is far more challenging than in a controlled cell environment. Despite over two decades of research and the development of various xenon host‐cages tested in vitro, only a few studies have demonstrated their application in vivo.

In 2017, Albert et al. injected CB[6] into rats via the tail vein and then allowed the rats to inhale HP ^129^Xe gas.^[^ ^194^ ^]^ The gas dissolved into the blood and interacted with the CB[6], enabling successful detection of CB[6] in the brain, heart, liver, kidneys, and aorta using Hyper‐CEST MRI. Although the used commercially available CB[6] lacks in targeting modifications for functional specificity, this work represents a milestone in the application of xenon hosts, marking the first detection at the in vivo level and setting the stage for future applications of ^129^Xe‐based probes.

Hyper‐CEST contrast relies on the reduction of the dissolved state signal after applying a selective saturation pulse. However, the concentration of dissolved ^129^Xe in tissues increases over time with continuous inhalation of HP ^129^Xe gas. Thus, reliable Hyper‐CEST contrast can only be achieved once a stable magnetization intensity of the dissolved ^129^Xe is established. Branca et al. outlined the experimental conditions necessary to obtain reliable contrast of Hyper‐CEST images in mice following intracorporeal injection of CB[6].^[^ ^195^ ^]^ Once the magnetization intensity of the dissolved ^129^Xe was stabilized, both on‐resonance and off‐resonance saturation frequencies were applied, and the Hyper‐CEST contrast map was obtained by subtracting the on‐resonance image from the off‐resonance image.

Another study reported an iron oxide nanoparticle that triggered HP ^129^Xe MRI signal‐off in the target lung cancer area.^[^ ^196^ ^]^ This negative contrast strategy leveraged the relaxation effects of paramagnetic substances to create negative contrast imaging. Iron tetraoxide nanoparticles, which accumulated at the tumor site through the EPR effect or active targeting after intravenous injection, generated a strong local magnetic induction gradient in the tumor region. This caused rapid dephasing of nearby transverse magnetization, resulting in a localized dark spot in the HP ^129^Xe MRI results.

## 6 Discussion and Future Perspective

MRI is one of the most powerful diagnostic tools for evaluating the structure and function of soft tissues and organs via direct visualization, whereas the inherent low sensitivity of conventional MRI hinders its further development. In commercialized MRI scanners, higher magnetic field and/or lower temperature are introduced to increase sensitivity. However, the required equipment is costly, and there are significant physical limitations that are difficult to overcome. According to the basic principles, the low proportion of efficient spin atoms that contribute MR signal leads to unfavorable sensitivity. Fortunately, hyperpolarization techniques could rebuild the nuclei spin of interest to a highly polarized state and enhance the signal intensity by several orders of magnitude.

For instance, in the case of hyperpolarized ^129^Xe, the angular momentum of photons is transferred to ^129^Xe atoms via a Ru atomic vapor, resulting in a 10 000–100 000 enhancement of the MR signal intensity. The introduction of HP ^129^Xe gas not only overcomes the challenges of imaging the lungs with traditional MRI, but more importantly, it offers a visualized method to quantify the localized lung structures and functions in a short scanning period. Briefly, HP ^129^Xe gas is inhaled to the lungs, and then passes through the trachea and bronchus, reaches the alveoli where a positive MR signal is generated to indicate the gas distribution in lungs. A new physiological parameter, namely, ventilation defect percentage (VDP), was introduced to quantify the ventilation function of lung and accurately evaluate the ventilation defect. Due to the restricted gas diffusion in the lungs, the multi‐*b*‐value DWI pulse sequence is able to detect the diffusion‐related parameters, such as mean chord length (*L*_m_), surface‐to‐volume ratio (SVR), and apparent diffusion coefficient (ADC), which quantifies the pulmonary microstructure in a noninvasive and nonradiative way. A variety of studies have confirmed that patients with some lung diseases display higher VDP values and microstructural abnormalities compared with health subjects, indicating the local ventilation function and microstructure could be directly visualized and quantified to evaluate the lung diseases by HP ^129^Xe MRI. Because of the favorable liposolubility, the inhaled ^129^Xe gases could further pass through the alveolar wall and dissolve in the blood, producing new MR signals that could be collected to quantify the local gas–blood exchange function in the lungs. By studying the dynamic transfer process between the dissolved ^129^Xe and ^129^Xe gas, some exchange‐related parameters, including exchange time constant (*T*), septal wall thickness (*d*), and RBC/TP, are quantified to reveal the damaged gas–blood exchange functions. Thus, the two main functions of lungs, gas–gas, and gas–blood exchanges, could be quantitatively evaluated in a single breath‐hold in HP ^129^Xe MRI. Moreover, the dissolved ^129^Xe in blood further binds with the red blood cells and travels to other parts of the body through the bloodstream, making it available for evaluation of other organs such as the brain and kidney.

To overcome the limitations in detecting specific molecules, functional host‐cages are employed to explore the application of ultrasensitive ^129^Xe MRI in the field of molecular imaging. The resulting molecular probe usually includes three parts, a host‐cage to capture the inert xenon, a targeting part to anchor specific molecules or cells, and a linking part to link the above two parts together. According to the structural differences, host‐cages that are able to capture xenon and generate corresponding MR signals are classified into two types, including molecular hosts and nano‐hosts. The inner diameter of molecular‐hosts is within a range of a few angstroms, a space that is slightly larger than xenon atom (4.3 Å). Therefore, one molecular host (such as cryptophane‐A, 5.4 Å) captures one xenon atom and then results in favorable MR signal that can be detected by conventional NMR/MRI methods. By contrast, the host space provided by nanocages is typically over 100 nm, a much larger space that is able to entertain thousands of xenon atoms. The exchange between the xenon atoms and nano‐hosts is greatly influenced by the diameter of the nanocavity, which can be characterized by MR signal‐enhancement techniques, such as CEST. Interestingly, recent studies revealed that on the condition of the similar nanoparticles’ diameter (≈100 nm), the pore diameter and the charge numbers in the skeleton structure collectively determined the affinity between xenon and MOFs.^[^ ^122^ ^]^ As a result, the ^129^Xe MR signals, represented as differentiations in chemical shifts and exchange rates, could be manipulated through tailoring the structure of the starting derivatives and metals of MOFs. Functionalization of these host cages through chemical modification, weak interaction and biological reactions further enables the targeting and/or fluorescent capabilities. Moreover, the linker between the cage and functionalized moiety could be designed as an environment responsible part, leading to activated or quenched signals when exposed to targeting areas. Through carefully designs, ^129^Xe‐based biosensors have shown good capabilities to targets, organelle and cells, as well as a detection limit at picomolar concentrations.

Biosafety is a primary concern that governs the in vivo and clinical applications of HP ^129^Xe MRI. As an inert gas and a component of the atmosphere, xenon exhibits minimal toxicity and is readily eliminated from the body.^[^ ^197^ ^]^ Previous studies have shown that the xenon elimination kinetics comprised a rapid initial phase, followed by a slower phase characterized by a first‐order kinetics and a xenon half‐life of 2.7 h.^[^ ^198^ ^]^ Clinically, xenon is used as an anesthetic, with a minimum alveolar concentration (MAC) ranging from ≈63% to 71%.^[^ ^199^ ^]^ In HP ^129^Xe MRI, the typical inhaled volume and the resulted alveolar concentration of xenon are significantly lower than the MAC required to induce anesthesia. The safety of HP ^129^Xe MRI has been demonstrated in healthy volunteers, children, and patients with various diseases.^[^ ^43^ ^]^ In addition, xenon does not participate in metabolic process^[^ ^200^ ^]^ within the body. Due to its low blood‐gas‐partition coefficient,^[^ ^201^ ^]^ xenon is rapidly eliminated from blood when alveolar concentrations decrease.^[^ ^198^ ^]^ These findings indicate that ^129^Xe can serve as a safe contrast agent, offering new avenues for MRI applications.

Overall, the HP ^129^Xe MRI with its ultrasensitivity, noninvasion, nonionizing radiation, deep tissue penetration, and short scanning period offers tremendous potential when used in bioimaging areas. This technique has shown promising results in applications ranging from structural and functional quantification of human lungs to brain function evaluation in animals, cellular detection, molecular analysis, and nanomaterial characterization.

Looking forward, we anticipate that ultrasensitive ^129^Xe MRI could become a powerful imaging modality for evaluating lung and brain diseases, as well as investigating the associated biological mechanisms. However, as an emerging technique, ^129^Xe MRI still faces fundamental and practical challenges that hinder its further development in biological imaging and the discovery of new applications. Nevertheless, numerous studies are expected to significantly advance and expand the use of ultrasensitive ^129^Xe MRI in the future, including but not limited to the following:

*(1) Increasing and maintaining high polarization levels of ^129^Xe*

High polarization level is the foundation for acquiring ultrasensitive MRI. Technically, the most challenging goal is to maximize the polarization level toward 100% hyperpolarization, where all the nuclei spins could contribute to the signal and then obtain the highest MR signal enhancement. So far, nine out of ten ^129^Xe atoms could be monitored under the highest polarization level of 90%. However, in the most commonly used flow mode during hyperpolarization, gas polarization for human ^129^Xe MRI remains below 50%. There is still room for improvement of the polarization to a higher level that leads to further increased MRI sensitivity.

Moreover, maintaining a high polarization level of HP ^129^Xe gas after production until it is inhaled or introduced into samples is crucial for achieving a high signal‐to‐noise ratio in NMR and MRI. Molecular motions, such as the intermolecular interaction and the collision between gas molecules and container walls, can retract HP ^129^Xe back to its thermal equilibrium state, leading to partial polarization loss. Storing HP ^129^Xe in liquid nitrogen can preserve its hyperpolarization state for 1–2 h. In short, preserving the high polarization level of HP ^129^Xe is closely related to the storage environment. Development of reliable theoretical models to study the hyperpolarization state of ^129^Xe atoms is essential in this respect. Besides the lower temperature that reduces the molecular motion, the physical properties of the container, which determine the collisional energy between HP ^129^Xe atoms and walls, are also crucial for maintaining of polarization level. Simultaneously, the magnetic field is another crucial factor influencing the polarization state. It is worth mentioning that a low magnetic field also accelerates the relaxation of HP ^129^Xe gas during the storage and transfer process before inhalation. Therefore, optimization of the magnetic field in the entire process including gas production, transfer, inhalation, and scanning, are crucial to the final MR signals.

Another common challenge in preserving the polarization level is the quenching effect of paramagnetic oxygen on the hyperpolarization of the inhaled ^129^Xe. In animal experiments, lungs are typically washed with oxygen and ^129^Xe separately before imaging to minimum the influence from the paramagnetic gas. However, in human studies, HP ^129^Xe gas is inhaled directly without any pretreatment. Consequently, residual oxygen in trachea, alveoli, and lung tissues may mix with the HP ^129^Xe gas, accelerating the relaxation attenuation of ^129^Xe. This reduction in steady‐state polarization levels can adversely affect signal acquisition. Adjusting the inhalation process to dilute the oxygen can help achieve higher levels of polarized ^129^Xe in the lungs, leading to better in vivo MRI results.

*(2) Reveling structural and functional changes of lungs to help evaluate diseases progression and treatment effectiveness*

HP ^129^Xe MRI is a powerful technique which resolves the problems of insufficient signal sources due to low gaseous density through enhancing the polarization level for tens of thousands of times, making it possible to visualize the whole process of gas transfer in the body, i.e., distribution of the inhaled gas in alveoli, exchange in lung tissues, binding with red blood cells, and delivery along the bloodstream to the heart, brain and other organs. Ongoing studies have illustrated that changes in structure and function observed by HP ^129^Xe MRI closely correlate to the progression of certain diseases.

Animal studies are typically performed before applying new methods to humans. By creating animal models for human diseases, researchers can investigate conditions that are challenging to study in humans due to the difficulties of controlling variables. These models facilitate a more accurate determination of the factors influencing certain diseases and the therapeutic effects of particular drugs. In gas MRI studies, the respiratory parameters of animals, such as the respiratory rate, inspiratory volume and pressure, are carefully controlled by respiratory system to eliminate individual differences and obtain high‐quality images. However, these results obtained under forced breathing may differ from those of free breathing. In addition, trachea injury caused by the employed invasive intubation complicates implement long‐term monitoring of the lungs. Therefore, measurements under natural conditions, such as spontaneous breathing, will provide a more representation of realistic physiological condition but present more technical challenges. Developing new breath‐control devices, such as respiratory mask, to balance the need for stable MR signal with biological safety considerations, would be beneficial for long‐period monitoring of animal models.

One of significant advancements is the ability to directly visualize local ventilation defects using HP gas MRI. This is particularly useful for locating specific areas before pulmonary resection or lung volume reduction surgery. Moreover, regional ventilation defects might be compensated by the healthy portions of the lungs, potentially causing misleading results in pulmonary function test (PFT) that appear to show normal lung ventilation. HP gas MRI is able to visualize, locate and quantify these regional ventilation issues, which may help reduce the false‐negative rate. The new technique also acids in evaluating the effectiveness of treatments for diseases involving ventilation defects, allowing for real‐time adjustment to therapy based on HP ^129^Xe MRI results.

Quantifying gas–blood exchange provides a new perspective to precisely assess lung function. For instance, HP ^129^Xe MRI reveals that the gas–blood exchange time of discharged COVID‐19 patients is relatively longer compared to uninfected health volunteers. This discrepancy explains why CT results might show their lungs have recovered, while patients still experience symptoms such as irregular breathing or dizziness. Studies in animal models indicate that radiation dose associated inflammation may accelerate gas transfer in lungs, suggesting that the gas‐blood exchange metric would potentially be important parameters for assessing and managing radiation‐induced lung injury. Furthermore, additional research needs to be carefully designed and conducted to establish more rigorous mechanisms for explaining the relationship between ^129^Xe MRI results and specific structural changes or physiological processes. For example, the differences of ^129^Xe MR signal oscillation in red blood cells has been observed in various diseases; however, the reasons behind this phenomenon are still unknown and require further investigation. In addition, HP ^129^Xe MRI is expected to provide more comprehensive information for clinician evaluating chronic inflammatory conditions of the respiratory system (such as COPD, asthma and pulmonary cystic fibrosis), as the accompanied airway disorganization, reconstruction, and mucous hypersecretion not only cause ventilation defect, but also affect gas–blood exchange and microstructure disorders of lungs.

Due to its noninvasion nature, lack of ionizing radiation, and ability to provide quantitative parameters on lung microstructure and function, HP ^129^Xe MRI holds significant promise for long‐time monitoring of lung diseases progression and treatment efficiency. To establish reliable reference intervals for diagnostic support, extensive data collection and analysis based on factors such as gender, age, weight, and region is essential. In addition, standardizing gas collection, MRI scanning, and data analysis processes will help streamline HP ^129^Xe MRI procedures, enhancing its potential for clinical use and widespread adoption.

Further studies on brain, heart, liver, and other organs with abundant blood flow using HP ^129^Xe MRI may become the next major focus, because the intensity and diffusion of ^129^Xe atoms delivered via the vasculature are directly related to the structure and function of local blood vessels. Although the signal enhancement method CEST is hardly applied to the lungs due to its motional features, exchangeable ^1^H has been used to study some endogenous molecules in the human brain. This approach could potentially be adapted for brain imaging using Hyper‐CEST. Moreover, exploration of fast signal acquisition and accurate reconstruction methods would further decrease costs, reduce usage thresholds and improve user experiences, making HP ^129^Xe MRI a promising tool for evaluating the structure and function of various organs.

*(3) Developing novel ^129^Xe‐based biosensors for in vivo molecular imaging*

Although functionalized hosts have been developed to capture the inert xenon, generate specific MR signal, and achieve low detection limits at picomolar levels, their in vivo applications as targeted ^129^Xe contrast agents remain challenging. Currently, CB[6] and iron oxide nanoparticles are the primary probes applied in HP ^129^Xe MRI for in vivo application. While CB[6] has been administered to mice via tail vein injection, its lack of targeting capabilities limits its use only for monitoring the distribution of this molecular host rather than providing specific signals for areas of interest. In contrast, iron oxide nanoparticles can target the tumor region and induce the relaxation of gaseous ^129^Xe signal, producing a black spot in HP ^129^Xe MRI that indicates nanoparticle's localization in the lungs. However, distinguishing the relaxed ^129^Xe signal from ventilation defects—both of which show similar absent gas signals in lung cancer tumors—remains challenging. Therefore, there is an urgent need to optimize and explore ^129^Xe “hosts” to enhance their signal stability, biosafety, and targeting ability for improved molecular imaging in vivo.

The lungs, with the highest concentration of HP ^129^Xe sources in the body, are potentially the most viable organ for achieving ultrasensitive molecular imaging in vivo. Moreover, highly polarized ^129^Xe could rapidly reach the lungs through inhalation, resulting in relatively minimal signal attenuation due to continuous relaxation over time. However, a short scanning period is essential to avoid motion artifacts caused by breathing during lung MRI, which complicates the application of indirect sampling Hyper‐CEST methods in vivo. Therefore, direct sampling MR methods are more feasible for lung imaging.

Existing^129^Xe host‐cages suitable for direct sampling include cryptophane and CB[6] molecular‐hosts, ZIF‐8 nanoparticles, and micro‐sized PFOB emulsions. Among these, molecular hosts generally exhibit the most stable ^129^Xe MR signal under physiological conditions, although modifications for targeting capability and biocompatibility present significant challenges. ZIF‐8 nanoparticles can achieve passive targeting of tumor regions through enhanced permeation and retention (EPR) effect, but they are prone to collapse under weak acidic or phosphorylated conditions, which may lead to signal quenching. Micrometer‐sized PFOB emulsions have lower in vivo targeting efficiency and require large doses. Consequently, the current ^129^Xe hosts are still inefficient in vivo and require innovative improvements.

For molecular hosts, loading them into suitable nanoparticle carriers may effectively address issues of biocompatibility and targeting. In addition, the EPR effect of nanoparticles can enhance their accumulation in rapidly proliferating regions. In this context, the loading rate of the molecular hosts is particularly crucial. It is also essential to maintain the interaction between the host‐cage and ^129^Xe atoms after loading and to ensure the stability of the hosted ^129^Xe MR signal in the targeted region to achieve effective in vivo molecular imaging. Besides ZIF‐8, some porous nanomaterials with well‐defined structures can generate their unique ^129^Xe MR signals using direct sampling methods in their solid state. However, when dispersed in aqueous solutions or other biological systems, only Hyper‐CEST signals may be detectable, or ^129^Xe MR signals may not be obtainable. This issue may arise because water molecules can enter the pores and alter the chemical environment that is originally designed to host ^129^Xe. Therefore, improving the hydrophobicity of their pore structures might enhance the ^129^Xe MR signal in porous nanoparticles. In addition, enhancing the biocompatibility of the outer surface of these nanoparticles is crucial to prevent aggregation and nonspecific binding in biological environments, thereby ensuring the acquisition of reliable and stable ^129^Xe MR signals.

For other relatively stationary organs (such as the brain and kidneys), motion artifacts are minimal, making Hyper‐CEST more feasible. However, hyperpolarized gases require time to be transported from the lungs to other organs via the bloodstream, during which polarization decay due to relaxation may lead to unstable ^129^Xe MR signals, posing challenges to indirect sampling methods based on signal decay like CEST. Therefore, developing rapid sampling and data processing methods to prevent signal decay is crucial for achieving in vivo Hyper‐CEST molecular imaging.

In conclusion, HP ^129^Xe‐based technology offers a feasible path to address low sensitivity and long scanning period issues in MRI, positioning it as a promising tool for next‐generation bioimaging. The advancement of HP ^129^Xe MRI hinges on the convergence and advancement of multiple disciplines, including atomic and molecular physics, chemistry, biology, materials science, and artificial intelligence. Overall, HP ^129^Xe MRI is anticipated to progress further in enhancing sensitivity, accelerating signal acquisition, image reconstruction, in vivo molecular imaging, and other aspects, ultimately delivering highly sensitive and visual insights for evaluating disease progression and studying molecular mechanisms in the lungs, brain, and beyond.

## Conflict of Interest

The authors declare no conflict of interest.

## Author Contributions

Y.Y., S.Y., and L.S. contributed equally to this work. Y.Y. conceived and designed the project, prepared, and revised the manuscript. S.Y. wrote the initial draft of Section 5. L.S. prepared the initial draft of Section 4. H.D. supported the citation of references. H.L., X.Z., and Q.G. assisted in proofreading the manuscript. X.Z. supervised and reviewed the manuscript.
